# Human pangenome analysis of sequences missing from the reference genome reveals their widespread evolutionary, phenotypic, and functional roles

**DOI:** 10.1093/nar/gkae086

**Published:** 2024-02-14

**Authors:** Zhikun Wu, Tong Li, Zehang Jiang, Jingjing Zheng, Yizhou Gu, Yizhi Liu, Yun Liu, Zhi Xie

**Affiliations:** State Key Laboratory of Ophthalmology, Zhongshan Ophthalmic Center, Sun Yat-sen University, Guangzhou, China; State Key Laboratory of Ophthalmology, Zhongshan Ophthalmic Center, Sun Yat-sen University, Guangzhou, China; State Key Laboratory of Ophthalmology, Zhongshan Ophthalmic Center, Sun Yat-sen University, Guangzhou, China; State Key Laboratory of Ophthalmology, Zhongshan Ophthalmic Center, Sun Yat-sen University, Guangzhou, China; Center for Precision Medicine, Sun Yat-sen University, Guangzhou, China; University of Wisconsin- Madison, WI, USA; State Key Laboratory of Ophthalmology, Zhongshan Ophthalmic Center, Sun Yat-sen University, Guangzhou, China; MOE Key Laboratory of Metabolism and Molecular Medicine, Department of Biochemistry and Molecular Biology, School of Basic Medical Sciences and Shanghai Xuhui Central Hospital, Fudan University, Shanghai, China; State Key Laboratory of Ophthalmology, Zhongshan Ophthalmic Center, Sun Yat-sen University, Guangzhou, China; Center for Precision Medicine, Sun Yat-sen University, Guangzhou, China

## Abstract

Nonreference sequences (NRSs) are DNA sequences present in global populations but absent in the current human reference genome. However, the extent and functional significance of NRSs in the human genomes and populations remains unclear. Here, we *de novo* assembled 539 genomes from five genetically divergent human populations using long-read sequencing technology, resulting in the identification of 5.1 million NRSs. These were merged into 45284 unique NRSs, with 29.7% being novel discoveries. Among these NRSs, 38.7% were common across the five populations, and 35.6% were population specific. The use of a graph-based pangenome approach allowed for the detection of 565 transcript expression quantitative trait loci on NRSs, with 426 of these being novel findings. Moreover, 26 NRS candidates displayed evidence of adaptive selection within human populations. Genes situated in close proximity to or intersecting with these candidates may be associated with metabolism and type 2 diabetes. Genome-wide association studies revealed 14 NRSs to be significantly associated with eight phenotypes. Additionally, 154 NRSs were found to be in strong linkage disequilibrium with 258 phenotype-associated SNPs in the GWAS catalogue. Our work expands the understanding of human NRSs and provides novel insights into their functions, facilitating evolutionary and biomedical researches.

## Graphical Abstract

**Graphical Abstract:**
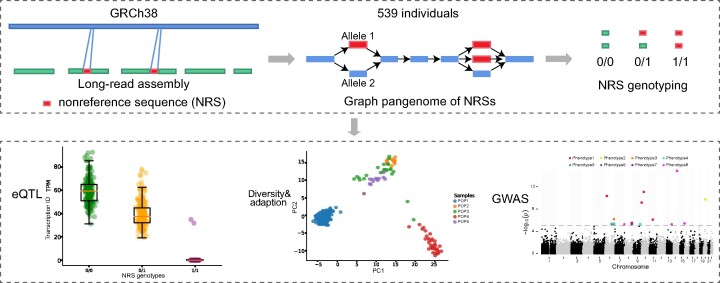

## Introduction

The human reference genome serves as a basis for aligning human sequences and has greatly progressed human genetic research (1). Despite its utility, the current reference genome, GRCh38, still contains numerous gaps (2). Another critical concern regarding the reference genome is its limited genetic representation. It is a linear genome generated from only about 20 individuals, primarily representing ancestral sequences from Africa (57%), Europe (37%) and East Asia (6%) (3). This inherent bias leads to an underrepresentation of the full spectrum of genetic diversity found in the global population, with a pronounced shortfall in capturing the diversity of Asia, which comprises 59.0% of the world's total population (https://www.worldometers.info/world-population/asia-population).

Nonreference sequences (NRSs) are sequences that are not present in the reference genome but are present in a subset of the population (3), which are also referred to as insertion (INS), a type of structural variation (SV). Some NRSs intersect genic regions or regulatory elements like enhancers, impacting gene structure or expression, which may be associated with human diseases or clinical phenotypes (4,5). In addition, some NRSs may contain the gene paralog (2) or have the transcription potential (6). In recent years, several studies have focused on NRSs. Duan *et al.* detected 29.5 Mb novel sequences in 275 genomes using HUPAN (7). Lee *et al.* uncovered 1696 NRSs from 2535 individuals through InserTag (8). And Chu *et al.* developed xTea to identify nonreference transposable element (TE) insertions from multiple platform data (9). Additionally, Meleshko *et al.* applied Novel-X to discover 18.2 Mb NRSs from 68 samples (10). Furthermore, *de novo* genome assembly in large-scale human sequencing projects have made progress in discovering NRSs (3,4,6). For instance, previous studies have reported the presence of 0.33 megabases (Mb), 29.5 and 46 Mb NRSs within Icelandic, Chinese and Swedish populations, respectively (7). However, these studies primarily relied on *de novo* assemblies of short or linked reads obtained from next-generation sequencing (NGS) platforms. Such approaches present challenges in accurately assembling segmental duplications (SDs), low-complexity regions, and regions exhibiting GC bias, particularly when conducting local assemblies of unmapped reads against the reference genome (8,11).

Long-read sequencing (LRS) platforms, including Pacific Biosciences (PacBio) continuous long read (CLR), PacBio high-fidelity (HiFi), and Oxford Nanopore Technologies (ONT), are renowned for their capacity to produce highly-contiguous *de novo* genome assemblies (12). The advantages of utilizing LRS for assembling repetitive regions render it particularly valuable in the discovery of large length NRSs, a challenge often encountered with SRS methods (3). Recent human genome assemblies, leveraging LRS data, exemplify this benefit. For instance, a Chinese genome (HX1) (13) and two Swedish genomes (14) demonstrated enhanced contiguity, boasting contig N50 lengths ranging from 8.3 to 9.5 Mb and revealing 12.8 and 12.2 Mb of NRSs per individual, respectively. Notably, marking a significant milestone in the two decades since the release of the first human genome, the completion of the CHM13 genome represents another remarkable achievement in the Human Genome Project (HGP). This endeavor added and refined 238 Mb of sequence (2). More recently, the Human Pangenome Reference Consortium (HPRC) (15) has proposed an ambitious project aiming to create a more sophisticated and complete human reference genome with a graph-based, telomere-to-telomere (T2T) representation to encompass global genomic diversity. The HPRC has recently unveiled its initial draft reference, including 47 phased, diploid assemblies (16). Furthermore, the Chinese Pangenome Consortium (CPC) has developed a Chinese pangenome, featuring a collection of 116 high-quality, haplotype-phased assemblies derived from 58 core samples representing 36 distinct Chinese ethnic groups (17). Additionally, Uddin et al. constructed the Arab pangenome reference from 43 individuals with diverse Arab ethnicities (18). Although the primary focus of these studies did not revolve around NRS discovery, they successfully identified numerous novel variants, haplotypes and alleles within structurally complex genomic loci. As more individuals with high-quality genomes are included, the human pangenome will offer a more comprehensive representation of global genomic variation, including NRSs. This invaluable genetic resource is set to play a pivotal role in advancing biomedical research and precision medicine (15,17).

While great progress has been achieved, our understanding of prevalence of NRSs within the human genome and among the human populations remains incomplete. Furthermore, the functional, evolutionary, and phenotypic significance of NRSs is still largely unknown. To tackle these pressing questions, we conducted a systematic identification of NRSs using data from 539 human genomes across five diverse populations, all sequenced using LRS technology. Subsequently, we constructed a graph-based pangenome. Our investigation encompassed a thorough characterization of NRS distribution within the human genomes, across various human populations, and even within nonhuman primates. We performed functional annotations to unearth insights into the roles of NRSs in evolution and disease. The utilization of a graph-based pangenome of NRSs not only offered enhanced representation of diverse populations but also yielded notable benefits in terms of read mapping rate and the detection of expression quantitative trait loci (eQTLs). Moreover, our exploration unearthed numerous NRSs with associations to local adaptation and phenotypic variations. This study provides a framework for constructing a graph-based pangenome of NRSs from large-scale LRS datasets. It furnishes crucial genomic resources and profound insights into the functions of NRSs, thereby facilitating advancements in evolutionary and biomedical research.

## Materials and methods

### Samples and datasets

In this study, we collected whole-genome LRS data for 539 samples from public databases. The genomes were *de novo* assembled from these LRS reads for each of the 539 samples, including 405 Chinese individuals sequenced by the ONT platform (19) and 134 individuals from diverse populations sequenced by the ONT, PacBio CLR and HiFi platforms (16,20,21). Of which, assemblies and corresponding sequences of 65 individuals were directly downloaded, including 47 high-quality phased, diploid assemblies from HPRC (16) (Supplementary Table S1). To ensure a high-quality genome assembly, we performed trimming the first 30 bases and last 20 bases of the ONT and PacBio CLR reads, which have relatively lower quality revealed in previous study (19), After trimming, any reads shorter than 500 bp were filtered out prior to assembly.

### *De novo* genome assembly of LRS datasets

Generating a high-continuity and more-complete genome assembly enabled more accurate detection of NRSs. To determine the sequencing depth required for reliable assembly metrics, we explored the correlation between key assembly metrics and different sequencing depths. Six samples with sequencing depths larger than 25-fold were randomly selected and their reads were downsampled to 2×, 4×, 8×, 12×, 15×, 18× and 22×. These downsampling reads were then used for *de novo* assembly using wtdbg2 (v2.5) (22) with parameters ‘-p 19 -AS 2 -s 0.05 -L 500’. For the *de novo* assembly with ONT data, wtdbg2 was used with the same parameters. To improve base accuracy of the assembly, the assembled contigs were polished using MarginPolish (v1.3.0) (23). The PacBio CLR data were assembled using wtdbg2 with parameters as before and were further polished using NextPolish (v1.4.0) (https://github.com/Nextomics/NextPolish) with the parameters ‘-r clr -sp’. The PacBio HiFi reads were assembled using hifiasm (v0.16.1-r375) (24) with default parameters.

We evaluated the completeness of assemblies and protein-coding genes using QUAST (v5.0.2) (25) after aligning assembled contigs to primary assembly genome GRCh38 excluding ALTs (13) along with corresponding gff annotation file (v95). To assess the base-level accuracy of the assembly generated from ONT reads, we used Inspector (v1.0.1) (26) to calculate the base quality value (QV) scores for randomly selected 10 samples. To further assess the assembly disagreement counts, as reported by QUAST, which mainly include instances of local misassembly and inconsistency, we employed the estimation methods proposed by Shafin et al. (23). For validation, 15-fold sequencing data from the ONT, PacBio CLR and HiFi platforms for HG002 was randomly selected and independently assembled using the strategy outlined in this study. The assemblies were them compared to the benchmark datasets for the HG002 genome v1.7, which represents a high-quality genome assembled using data from multiple platforms (27).

### NRS detection

**Figure 1.**
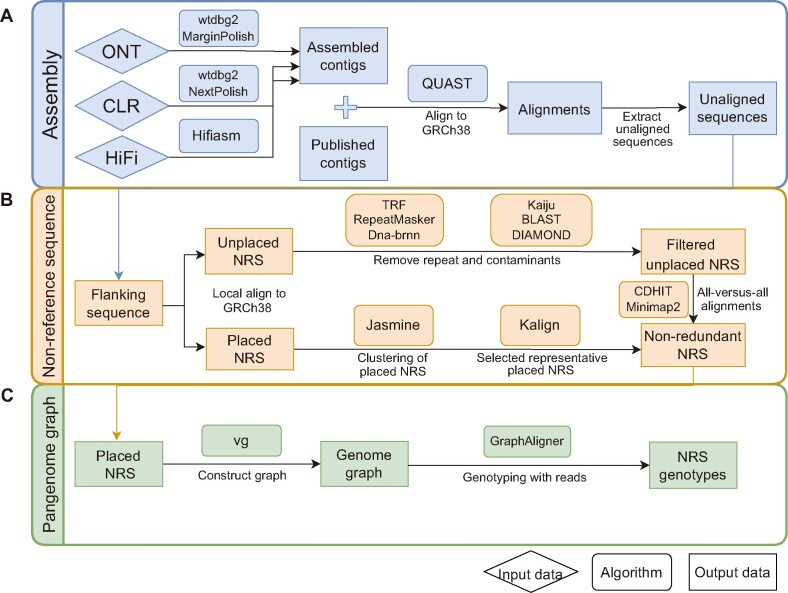
Schematic representation of GraphNRS. (**A**) Long-read sequencing data from different platforms are *de novo* assembled and polished. (**B**) The NRSs are anchored to GRCh38. Placed NRSs are clustered to select the representative NRSs, and unplaced NRSs are clustered after filtering out contaminants and centromeric repeats. Then, we merge the placed and the unplaced NRSs to obtain the nonredundant NRSs of the whole population. (**C**) vg is used to construct the graph pangenome, and NRS genotyping is performed for each NRS of the individual.

We applied a hierarchical strategy to extract NRSs (Figure 1A). For each individual, we initiated the NRSs extraction process from unaligned contigs employing QUAST (7) using command ‘quast –no-gc –no-plots –no-html –no-snps –min-contig 1000 -o output -r ref_genome.fa -g ref_genome.gff -t threads assembly_genome.fa’. It is important to note that we used the reference GRCh38.p13, which contained patches scaffolds, alternate loci and mitochondrial sequence, Epstein-Barr virus sequences (AJ507799.2) and decoy sequences (GCA_000786075.2), for subsequent alignment, thereby confirming the presence of sequences absent from the reference genome. We then aligned the LRS reads to the assembled contigs, and estimated their depths using mosdepth (v0.2.5) (28). We filtered out NRSs that were designated as either collapsed or with low read depth, considering depth values that exceeded three times or were less than one-third of mean depth for the corresponding individual.

Heterochromatic and centromeric regions are known to consist of tandem repeats Hsat2,3 and Alpha satellites, which contributed to most of gaps of genome assembly (29,30). To specifically identify and remove Hsat2,3 and Alpha satellites, we employed RepeatMasker (v4.0.9) (http://www.repeatmasker.org) and dna-brnn (v0.1-r65) (31). Regions that were masked were removed if they constituted at least 80% of total sequence length.

Mosè Manni *et al.* reported that some previously published human pangenome studies had overestimated the number of NRSs due to contamination from bacteria associated with original samples or introduced during sequencing experiments. In this study, we used the method recommended by Mosè Manni et al. (32) to remove contaminants (Figure 1B). First, we masked low-complexity and repetitive regions using RepeatMasker and TRF (v4.09) (33), based on Dfam (v3.0) (34) and RepBase (v2018-10-26) (35). Then, we used Kaiju (v1.7.3) (36) with parameters ‘-t nodes.dmp -f kaiju_db_nr_euk.fmi -i non_ref.fa -a mem -z threads -o kaiju.out –v’ to classify the remaining unaligned sequences, which had a good recall rate to detect more divergent sequences at amino acid level (32). Through aligning above sequences against the pre-formatted ‘nr + euk’ database (v2019-06-25), which contained protein sequences from bacteria, archaea, viruses, fungi, and microbial eukaryotes, we obtained a taxonomic classification of each unaligned sequences based on the continuous alignment with at least 100 amino acids and marked the sequences with label of ‘non-chordate’. Furthermore, we searched the sequences against the nr and nt (v2020-01-09) databases using DIAMOND (v0.9.21) (37) and BLAST (v2.10.0+) (38), respectively. Based on the alignment and taxonomic classification, we retrieved the sequences labelled with chordate which were originally labelled as non-chordate by Kaiju. To ensure accurate NRSs, we considered any sequences labelled as non-chordates to be contaminants and removed them from further analysis.

To study the impact of population size on the nonredundant NRS count, we randomly selected the individuals in this study. Based on previous studies that reported higher genetic diversity and more unique SVs among Africans (AFRs) (20), we divided all individuals into two categories: non-AFRs and AFRs. The process was reported 10 times, each time adding one individual. The NRS count was determined by taking the average of the selected individuals’ NRS counts.

To assess the efficacy of identifying NRSs against the more complete genome T2T-CHM13, we randomly selected 23 samples from five diverse populations. Employing the same pipeline as in GRCh38, we detected NRSs for each sample and them merged all NRSs from these samples into a unified callset. Genomic coordinates of NRSs in T2T-CHM13 were converted to GRCh38 using LiftOver (https://liftover.broadinstitute.org/) with the corresponding chain file (https://hgdownload.soe.ucsc.edu/goldenPath/hs1/liftOver/). Considering that the NRS lengths of the two genomes overlap by at least half at the same position, we posit that they represent the same NRS. Subsequently, the ratios of overlapped NRSs across diverse populations were calculated.

### Anchoring and validation of NRS

In order to accurately anchored the NRSs, we extracted the two flanking sequences of the identified NRSs, each with a length of 1 kilobase (kb), a size for long-read alignment also employed in a previous study (39). Subsequently, we individually aligned these sequences to the reference genome GRCh38 using AGE (v0.4) (40). If the alignment length of the flanking sequence was more than 500 bp, and the two end coordinates were less than 20 bp apart, the original sequence was considered successfully anchored to the reference genome. The locations of placed NRSs relative to the reference genome were plotted using in-house script modified from RIdeogram (v0.2.2) (41). If either upstream or downstream sequences could not be successfully aligned to the reference, the sequences were regarded as unplaced. To further ensure reliable unplaced sequences, the unplaced sequences and contigs were realigned to genome using minimap2 (v2.24) (42). Any sequence that aligned with identity ≥90% and length coverage ≥ 80% of total length was removed. To further filtered out unplaced sequences, we applied filters based on previous strategy: (i) sequences with >80% masked bases according to TRF were removed and (ii) sequences with >80% combined masked bases annotated as satellites, simple repeats, and low complexity regions by RepeatMasker were removed (5). The remaining unplaced sequences were retained for further analysis.

To confirm the accuracy of the NRSs obtained using our strategy, we compared the NRSs extracted from a *de novo* assembly of HG002 using 15-fold ONT reads with those extracted from the HG002 assembly that was used as a GIAB benchmark dataset (27). In addition, we *de novo* assembled the genomes of 10 samples from this study that were sequenced using PacBio HiFi reads at an average depth of 12× (19), and extracted the NRSs. We then evaluated the number of ONT-derived NRSs from these 10 samples that were validated by the NRSs from of the PacBio HiFi data assemblies. The NRSs supported by at least two reads were considered validated.

To analyze the hotspot of NRSs in the genome, we applied the function ‘hotspotter’ from the primatR package (https://github.com/daewoooo/primatR) with parameters ‘bw = 200000, num.trial = 1000’. We calculated a *P*-value by comparing the density of NRS locations with the density of a randomly sampled subset of the genome.

### Nonredundant NRS of the population

We applied Jasmine (v1.1.0) (43) to generate nonredundant sequences for both placed and unplaced NRSs. For the placed NRSs, we first calculated the median anchored positions within the reference genome for both upstream and downstream regions of these sequences. Subsequently, we employed Jasmine to combine the placed NRSs using parameters ‘–output_genotypes –ignore_strand –keep_var_ids’. To constrain the distance between each paired sequence, we limited it to 250 bp for all pairs within each cluster. Next, we conducted multiple sequence alignments using Kalign (v3.3) (44), applying a scoring system of match (+2), mismatch (−1) and gap opening (−0.5). The sequence with the highest score was chosen as the representative sequence. For the unplaced NRSs, we performed all-versus-all alignments using minimap2 with the parameters ‘-DP -t threads unplaced.fa unplaced.fa > aligned.paf’. The alignment pairs meeting the criteria of an alignment length ≥200 bp and sequence identity ≥90% were retained for downstream analysis. Sequence that did not intersect with others were considered nonredundant. If the alignment length covered at least 80% of one sequence, the shorter one was removed. Lastly, we excluded the unplaced sequences that existed in only one individual. Consequently, we obtained the nonredundant NRSs representing the whole population. We divided the NRSs into four categories based on their allele frequency (AF) in the whole population: singleton (allele count = 1), polymorphic (allele count ≥ 2 and AF < 0.5), major (AF ≥ 0.5 and AF < 1) and shared (AF = 1). Five trios were included in this study. When calculating the NRS AF in the population, we excluded the offspring of these trios.

### Graph-based pangenome of NRS and genotyping

To determine the genotypes of the NRSs, we first constructed a graph-based pangenome by merging the placed NRSs with the reference genome GRCh38 using vg toolkit (v1.33.1) (45) with parameters ‘vg construct -a -f -p -S -m 32’. The unplaced NRSs were added to the end of pangenome. To enable robust downstream analyses, we performed accurate genotyping of the placed NRSs using the constructed pangenome graph. We aligned the long reads of each sample to the pangenome graphs using GraphAligner (v1.0.13) (46), followed by graph-based genotyping of all NRSs for each sample using vg. The long reads of each individual were first aligned to the graph reference with GraphAligner using parameter ‘-x vg’. The NRSs were then genotyped according to the long-read alignment with vg. If the NRSs were extracted from an individual, the genotype for that individual would be ‘0/1’ or ‘1/1’. The recall rate of the genotyping was evaluated based on presence and absence information of NRSs. We estimated the Mendelian error rate for five trios.

### Comparison of NRS to other human genomes and pangenomes

**Figure 2.**
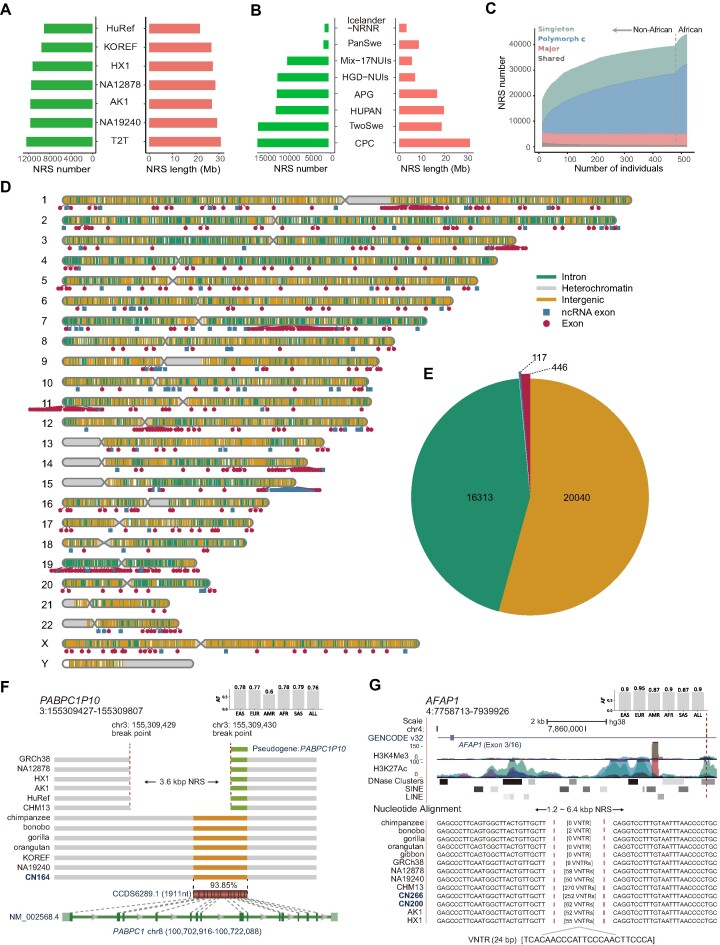
Characterization of NRSs for the whole population. (**A**) Overlapped NRSs of this study to the different human genomes. (**B**) Overlapped NRSs of this study to the different human pangenomes. (**C**) The growth of the NRS number with an increase in sample size. The growth before the vertical dotted line is for non-Africans, and the growth after the vertical dotted line is for Africans. Four categories based on the allele frequency (AF) are shown. (**D**) Locations of the nonredundant NRSs against the reference genome GRCh38. The gray, orange and green vertical lines on the chromosomes represent NRSs located in heterochromatin, intergenic and intron regions, respectively. The blue and red dots represent the NRSs located in exons of noncoding and protein coding genes, respectively. (**E**) No. of nonredundant NRSs intersected with different gene types. (**F**) A 3.6 kb NRS anchored to the left end of pseudogene *PABPC1P10* in nonhuman primate and human genomes. The green and orange bars represent *PABPC1P10* and the region with high identity to the CDS of *PABPC1*, respectively. The sample name in blue indicates the genome assembly generated in this study. The bar chart located in the upper right corner represents the AF of NRS. (**G**) An NRS composed of a VNTR with a 24-bp repeat unit present in nonhuman primate and human genomes. The bar chart located in the upper right corner represents the AF of NRS.

Human assembly genomes from various ancestries backgrounds, including T2T-CHM13 (2), HX1 (13), AK1 (47), KOREF (48), HuRef (49), NA12878 (12) and NA19240 (50), have been documented (Figure 2A). In addition, various human pangenomes, such as the Chinese HUman Pangenome Analysis (HUPAN) (7), African pangenome (APG) (51), Icelander nonrepetitive NRSs (Icelander-NRNR) (4), 1000 Swedish genomes (PanSwe) (52), Swedish genomes (TwoSwe) (14), Mix-17NUIs (6) and HGD-NUIs (53), have been reported by large-scale whole-genome sequencing using NGS data (3) (Figure 2B). We also downloaded the Chinese pangenome reference (17) (CPC.Phase1.CHM13v2-full) from CPC website (https://pog.fudan.edu.cn/cpc/#/data). We extracted the sequences of INSs (≥50 bp) using vg deconstruct with default parameters. Then, we utilized LiftOver to convert the genomic coordinates of T2T-CHM13 to GRCh38 based on the corresponding chain file. Using the same approach, we compared the NRSs identified in this study to the inversions from several other published SV datasets, including 405 Chinese (https://ngdc.cncb.ac.cn/gvm/getProjectDetail?project=GVM000132) (19), 3622 Icelanders (https://github.com/DecodeGenetics/LRS_SV_sets/blob/master/ont_sv_high_confidence_SVs.sorted.vcf.gz) (54), HGSVC2 (http://ftp.1000genomes.ebi.ac.uk/vol1/ftp/data_collections/HGSVC2/release/v2.0/integrated_callset/variants_freeze4_sv_insdel_alt.vcf.gz) (20) and HPRC pangenome (https://s3-us-west-2.amazonaws.com/human-pangenomics/pangenomes/freeze/freeze1/minigraph/hprc-v1.0-minigraph-grch38.bb.bed.gz) (16). The nonhuman primate genomes, including chimpanzee, bonobo, gorilla, Sumatran orangutan, Bornean orangutan and siamang gibbon (https://github.com/marbl/Primates), were also used to detect overlapped NRSs identified in human genomes. Reciprocal alignments between our NRSs and these assemblies were carried out using minimap2 with the parameters ‘-x asm20 -t threads nonref.fa genome.fa > aligned.paf’ and only alignments with an overall identity ≥90% and coverage ≥80% of the NRSs were retained (51). An alignment was considered as reliable if it had a length ≥200 bp with an aligned identity ≥90% and an aligned coverage ≥80% of this sequence.

### Annotation of nonredundant NRSs

To reduce the false-positive rate in protein-coding gene annotation, we first masked the repeat sequences as ‘N’ using method mentioned above. Subsequently, we downloaded all expressed sequence tags (ESTs) of human from Ensembl and human protein sequences from NCBI (v2020-03-17). To eliminate redundancy within both ESTs and protein sequences, we independently employed CD-HIT (v4.8.1) (55) with the following parameters ‘cd-hit-est -i est.fa -o est.cdhit.fa -c 0.9 -n 8 -d 0 -M 0 -T threads’ for ESTs and ‘cd-hit -i protein.fa -o protein.cdhit.fa -c 0.9 -n 5 -d 0 -M 0 -T threads’ for protein sequences. Protein-coding genes within the nonredundant NRSs were predicted from the repeat-masked NRSs using MAKER2 (v2.31.1) (56). During this process, SNAP (v2006-07-28) (57) underwent two rounds of training based on the EST sequences. A*b initio* gene prediction was performed using Augustus (v3.3.3) (58) with the human model. Transcripts with a length >150 bp were retained, and models with an annotation edit distance (AED) ≤0.5 were used to remove poor-quality models (59). To ensure the reliability of our predicted genes, we conducted blastn and blastx searches against the complete sequences in NCBI nt and nr databases, respectively. This was done to ascertain whether the gene was conserved in other primates or elsewhere in the mammalian genome. The thresholds of *e*-values for blastn and blastx were set at 1 × 10^−15^ and 1 × 10^−7^, respectively. We then annotated the domains that matched either the Pfam protein families database (60) or the NCBI Conserved Domain Database (CDD) (61). The predicted genes without intron was regarded as pseudogenes.

### NRS associated eQTLs

To evaluate the impact of NRSs on gene expression levels, we utilized the data from 451individuals of the Genetic European Variation in Disease (GEUVADIS) consortium (62). For the short-read sequences obtained from Illumina platform, we employed vg giraffe for read alignment to the graph genome with parameters ‘-p -b default –rescue-algorithm dozeu’. Then, vg was used to genotype the NRSs according to the short-read alignment with parameters ‘vg pack -Q 0; vg call’. Through our evaluation on the HG002 dataset, we found that the overall sensitivity and specificity of NRS genotyping using SRS data were 0.69 and 0.76, respectively. These values were slightly lower than those in non-tandem repeat regions (0.81 for sensitivity and 0.86 for specificity). We obtained the genotyped single nucleotide polymorphisms (SNPs) of GEUVADIS consortium (62) with GRCh37 as the reference, which we subsequently converted to GRCh38 using LiftOver. We explored the association between gene expression levels and NRSs within a 1 Mb window centered around the gene's transcription start site (63). Based on the graph pangenome of NRSs constructed in this study, we genotyped the NRSs for 451 individuals from whole-genome NGS data. The resulting NRS genotypes have been made publicly accessible via a GitHub repository (https://github.com/xie-lab/GNRS/tree/main/data). From this dataset, we selected 7244 genotyped NRSs with a minor allele frequency (MAF) >0.05 after filtering out multi-allelic NRSs. Subsequently, we quantified the transcript-level expression based on RNA data using the graph-based method facilitated by vg mpmap and rpvg (https://github.com/jonassibbesen/rpvg) according to the previously estimated pipeline (64). To identify NRS-associated eQTLs, we performed principal component analysis (PCA) on the genotype matrix of NRSs. We then conducted an association analysis between transcript-level expression and NRSs genotypes within 1 Mb window using fastQTL (v2.165) (65). Finally, we used the Benjamini-Hochberg procedure to identify all NRS- eQTL pairs at 5% false discovery rate (FDR). The NRSs were annotated using Ensembl Variant Effect Predictor (VEP) (v103.1) (66), treating NRSs as insertions. The classification of annotations adheres to the methodology outlined by Sherman *et al.* (51).

### Population stratification and local adaption

To determine the population stratification among AFRs, Americans (AMRs) and East Asians (EASs), we employed EIGENSOFT (v7.2.1) (67) to conduct a PCA using 4685 NRSs (MAF > 0.05) that met the Hardy-Weinberg equilibrium (HWE) criteria (p-value threshold of 0.0001). Subsequently, we calculated population branch statistics (PBS) for subpopulations using PBScan (v2020-03-16) (68). Only NRSs that exhibited polymorphic within subpopulations were considered for further analysis. A rank of 99.9% was used as the threshold for departure from neutrality. We performed PBS for AFR, AMR and EAS using 13518 NRSs (MAF > 0.01). In this study, over-representation of EASs had the potential to introduce ancestry bias and affect result accuracy. To mitigate this concern, we downsampled the number of EASs to 40, repeated 10 times. The identified sites with PBS scores in the top 0.1% in at least 7 of the 10 downsampled iterations were considered candidate loci under selection. SVs exceeding the PBS threshold within a continuous 1 Mb were combined as independent single signal. To ensure the reliability of our loci, we implemented a filtering step to mitigate the potential influence of batch effects, which may arise when data is collected from diverse platforms. We conducted a chi-squared test on the NRS genotypes within each population, such as those from PacBio CLR and HiFi in AFRs, and those from ONT and PacBio HiFi in EASs. Subsequently, the *P* values were corrected using the Benjamini–Hochberg method, and loci with a corrected *q* value <0.05 were identified as susceptible to batch effects and thus excluded from the PBS results.

### Genotype and phenotype association analysis

In this study, we analysed the association between clinical phenotypes and genetic variations using 5643 genotyped NRSs with a MAF >0.05 in 327 individuals. A genome-wide association study (GWAS) was performed using PLINK (v1.90b4) (69) with linear regression under an additive genetic model for the quantitative traits. Age, sex, body mass index (BMI), and the first two principal components were included as covariates, except for BMI when performing the GWAS, which was excluded from the covariates. Logistic regression was used to test the association in a case-control analysis. The significant threshold was set to be 8.9 × 10^−6^ after applying Bonferroni correction (0.05/5643) (70).

### SNP detection and linkage disequilibrium analysis

In our previous study involving 405 samples (19), we employed longshot (v0.4.1) (71) to detect SNPs across the genome for each sample, utilizing BAM files aligned to the GRCh38 reference. To obtain high-quality SNPs, we applied a filter requiring a minimum of 8 supported reads and a minimum quality score of 20. Additionally, 105 440 SNPs associated with the phenotypes in GWAS catalogue (r2020-03-08) (72) were extracted as our target SNP dataset. Subsequently, we constructed the matrix representing the target SNP and the 405 samples, coding missing genotypes at the target loci as ‘0/0’ for the homologous reference. Finally, we used PLINK to calculate the linkage disequilibrium between the target SNP dataset and NRSs detected in this study.

### Statistical analysis

For each NRS, we performed Fisher's exact test to evaluate the Hardy–Weinberg *P* values. NRSs with a *P* value <0.0001 were considered to have failed the HWE, as described in previous study (73). We conducted a Wilcoxon signed-rank test to compare the read mapping rates derived from linear reference genome and pangenome. Additionally, a chi-squared test was performed on genotypes from different platforms within the population, and *P* values were corrected using the Benjamini–Hochberg method. To perform gene ontology (GO) enrichment analysis, we utilized GO annotation files obtained from Enrichr website (https://maayanlab.cloud/Enrichr/) (74). Fisher's exact test was employed for GO enrichment analysis, and the resulting *P* value were subjected to correction using the Benjamini–Hochberg method. The statistical tests used in the analysis are described throughout the article and in the figures. In the box plots, the upper and lower hinges represented the first and third quartile. The whiskers extended to the most extreme value within 1.5 times the interquartile range on either end of the distribution, and the center line represented the median.

## Results

### NRS discovery

To identify reliable NRSs from *de novo* assemblies of LRS data, we developed a pipeline consisting of two crucial steps: (i) the *de novo* assembly of genomes and (ii) the extraction of high-confidence NRSs (Figure 1A, B and Materials and methods). Furthermore, we integrated a critical step for constructing a graph pangenome of NRSs, which we aptly named GraphNRS.

To achieve a highly contiguous genome assembly, we first estimated the required sequencing depth for long reads. We assembled the individual genome based on sequences randomly extracted from six ONT datasets. Our findings demonstrated that the cumulative length of contigs assembled using a 12-fold depth data was comparable to that obtained from a 25-fold depth dataset (Supplementary Figure S1A). In addition, our assemblies achieved N50 lengths exceeding 13 Mb with a 15-fold depth dataset (Supplementary Figure S1B). To evaluate the accuracy of our assembly strategy, we applied it to the 15-fold HG002 datasets generated by ONT, PacBio CLR and HiFi, respectively. We compared these assemblies to the benchmark data described by Shumate et al. (27). The results revealed that the base-level error rates for ONT, PacBio CLR and HiFi were 0.93%, 0.49% and 0.12%, respectively. The assembly disagreements were minimal, with counts of 152, 132 and 182, respectively (Supplementary Table S2). The errors of the assemblies across the three platforms were either lower or comparable to those of the assembly with more than 50-fold ONT reads (135 for assembly disagreements) (23). In conclusion, our assembly strategy yields reliable genome assembly using sequencing data with approximately 15-fold depth.

In this study, we collected public datasets comprising 539 samples, mainly including a cohort of 405 Chinese individuals (19), 25 Tibetan and Han Chinese (21), 52 individuals in HPRC (16) and 32 individuals in Human Genome Structural Variation Consortium (HGSVC) (20). After quality assessment, we individually *de novo* assembled 473 genomes sequenced by LRS platforms with an average sequencing depth of 19.6-fold (Supplementary Table S1, Methods). To further improve the assembly quality, we polished the assemblies except for those derived from PacBio HiFi datasets. Additionally, we downloaded 66 publicly available genomes sequenced by LRS platforms with quality assessment. In total, we obtained 539 assemblies, where 431, 39 and 69 were obtained from the ONT, PacBio CLR and HiFi platforms, respectively (Supplementary Table S1). The average length of 539 assemblies was 2826 Mb, successfully recovering 93.9% of GRCh38 and 93.1% of the protein-coding sequences (Supplementary Figure S2A). These assemblies produced high-contiguity contigs, with an average N50 length of 16.3 Mb (Supplementary Figure S2B), which was much longer than the previously published genomes HX1 (8.3 Mb) (13) and NH (3.6 Mb) (75), both of which were sequenced on PacBio CLR platform. The average base-level QV across 10 randomly selected assemblies was 29.5, with scores ranging from 28.1 to 32.4 (Supplementary Table S3), which was comparable to base quality scores of assemblies reported by Shafin et al. (QV = 30) (23). In addition, the draft assembly had a high level of macrosynteny with the reference genome GRCh38, confirming the accuracy of our assemblies (Supplementary Figure S3). Consistent with a previous study, we found that LRS-based assembly detected errors that occurred in GRCh38, which was mostly based on BAC sequences and thus might result in multiple gaps and errors in the regions of scaffold switch-points (63). For instance, in the switch-point of two original BACs, RP4-783C10 and RP11-109P14, a 2.4 kb sequence was missed in GRCh38 (Supplementary Figure S4A), and the missing sequence could be recovered in the assemblies in our study (Supplementary Figure S4B).

We successfully extracted high-confidence NRSs from the assemblies using a hierarchical method (Figure 1 and Materials and methods). First, we aligned the assembled contigs to the reference genome GRCh38 and initially extracted an average length of 17.0 Mb raw NRSs. To obtain high-confidence NRSs, we removed contaminants, satellite sequences around the centromeric regions, contigs with ultralow or ultrahigh depths and unplaced singleton NRSs (Materials and methods). To evaluate the reliability of our identified NRSs from the assemblies of ONT reads, we leveraged assemblies generated with PacBio HiFi reads. The presence of NRSs was assessed in 10 samples in this study as well as HG002 (Supplementary Table S4), which were sequenced by both ONT and PacBio HiFi (27). We found that on average 88.9% of NRSs (ranging from 86.9% to 90.3%) could be validated by PacBio HiFi data (Supplementary Table S4), showing the reliability of the extracted NRSs. Subsequently, we applied our method to the 539 assemblies. In total, we identified 5.1 million high-confidence NRSs for all the samples, with each individual having an average length of 6.3 Mb NRSs (Supplementary Figure S5A). After merging and removing redundant NRSs, we obtained 45284 nonredundant NRSs with a cumulative length of 59.7 Mb and an N50 length of 3.7 kb (Supplementary Figure S5B).

### NRSs in the human genome

Our *de novo* assembly strategy successfully identify both placed and unplaced NRSs relative to GRCh38, with 36853 (spanning 27.3 Mb) placed and 8431 (32.4 Mb) unplaced NRSs (Table 1 and Supplementary Table S5). The average length of unplaced NRSs was longer than that of placed NRSs (3844 bp versus 740 bp, Table 1). The substantial difference can be attributed to the fact the unplaced NRSs often reside in complex regions such as SDs where GRCh38 exhibits limited sequence representation. The NRSs were compared to the previously published genomes and pangenomes (3). Among the five human genomes we compared, the most overlapped NRSs were found in currently the most complete genome, T2T-CHM13 (2), including 12377 NRSs with a total length of 30.7 Mb (Figure 2A and Supplementary Table S6). Among the pangenomes, the Chinese pangenome reference had the most overlapping NRSs (17 082) with our set, with a total length of 31.1 Mb. This is attributable to the 116 high-quality and haplotype-phased assemblies from 58 individuals representing 36 minority Chinese ethnic groups (17) (Figure 2B). Additionally, 23 202 NRSs with a total length of 12.1 Mb overlapped with insertions from several large-scale SV datasets or pangenomes (Supplementary Table S7). In total, we confirmed 31 843 (70.3% of total) NRSs with a cumulative length of 44.2 (74.1%) Mb in the previous datasets (Table 1). The substantial proportion of novel NRSs (29.7%) showed that our study greatly expanded our current knowledge of the human genome.

**Table 1.** Summary of the nonredundant NRSs

Category	No. of NRSs	Total length (bp)	Average length (bp)	NRSs per sample	Samples per NRSs	No. of recovered NRSs^a^	Length of recovered NRSs (bp)
Placed	36 853	27 259 120	740	8577	125	26 782	15 222 941
Unplaced	8431	32 409 688	3844	834	53	5061	28 969 342
Total	45 284	59 668 808	1318	9410	112	31 843	44 192 283

^a^The number of nonredundant NRSs intersected with the previously published genomes and pangenomes (details in Materials and methods).

We observed that NRSs were nonrandomly distributed in the genome. Of the 36 853 placed NRSs, 8307 (22.5%) were located in the last 5 Mb of chromosome arms (spanning 240 Mb), showing an enrichment at the end of these arms (odds ratio = 2.8, *P* = 8.8 × 10^−68^, Fisher's exact test). In addition, we identified 144 hotspots spanning 141 Mb of the genome (Supplementary Table S8). Of these, 112 (77.8%) were located in SDs as nonallelic homologous recombination associated with SDs serves as a crucial mechanistic catalyst for the NRS hotspots (76).

Among all the NRSs we identified, 78.5% constituted repeat sequences (Supplementary Table S9). The percentage of repeat NRSs falls within the range observed in some previous studies (75.0–88.6%) (7,14). The repeat NRSs encompassed various types of repeat elements, including variable number tandem repeats (VNTRs, 17.8%), short tandem repeats (STRs, 11.4%), short interspersed nuclear elements (SINEs, 14.5%) and long interspersed nuclear elements (LINEs, 15.9%) (Supplementary Table S9). The enrichment of repeat elements can likely be attributed to the fact that these NRSs originate from flanking regions with low-complexity sequences in the genome, where repetitive sequences have a tendency to expand and diversify.

### NRSs in human populations and nonhuman primates

In this study, various NRSs were examined, and their distribution is as follows: shared NRSs (AF = 1) accounted for 1.1%, major NRSs (1 > AF ≥ 0.5) constituted 10.9%, polymorphic NRSs (0.5 > AF but not singleton) made up the majority at 74.8%, and singleton NRSs (occurred in one sample) comprised 13.2%. It was noteworthy that the low frequency NRSs (AF < 0.1) constituted a large proportion of the NRSs, accounting for 69.4% (Supplementary Figure S6A). Among all novel NRSs, 77.3% had an AF less than 0.1, highlighting the value of using a large-scale diverse population to discover novel NRSs. The novel NRSs exhibited enrichment in coding sequences (CDS) (odds ratio = 2.0, *P* = 1.5 × 10^−12^, Fisher's exact test) of protein-coding genes. This suggests a higher likelihood of novel NRSs possessing genetic functions. To examine the distribution of NRSs in regional populations worldwide, the NRSs were compared across five major populations: EAS, AFR, South Asian (SAS), European (EUR) and AMR. Previous studies showed that the AFR population had more SVs compared to non-AFRs due to their large genetic diversity (63). Here we found that AFRs had a significantly larger number of NRSs than non-AFRs (*P* = 1.5 × 10^−30^, two-tailed *t* test, Supplementary Figure S6B). Out of the 45284 NRSs, 38.7% (17521) were common in all five populations, and 35.6% (16122) were population specific (Supplementary Figure S6C). After excluding the NRSs from CPC, the proportion of population-specific NRSs decreased to 14.2% (6438) (Supplementary Figure S6D). Among the novel NRSs, 12431 NRSs (92.5% of novel NRSs) were identified in the EAS population, with 4875 being specific to EAS (Supplementary Figure S6E). While the CPC made significant contributions, the substantial sample size of our study allows for the identification of a greater number of novel NRSs.

To determine the requisite sample size for capturing a significant portion of the NRSs within the population, we analysed the growth of the NRS numbers relative to the population size. Given the considerably higher number of NRSs in the AFR population compared to non-AFR populations, we conducted separate analyses for both groups. We observed that the number of shared and the major NRSs remained stable in non-AFRs and did not change after including AFRs (Figure 2C). In contrast, the number of polymorphic NRSs and the singletons gradually increased in the non-AFRs and accelerated with a higher positive slope after including AFRs (Figure 2C). This suggests that our study effectively captured the vast majority of the shared and the major NRSs for both non-AFRs and AFRs. However, to encompass the polymorphic NRSs and singletons, especially among AFRs, a larger sample size is necessary.

To trace the origin of the NRSs, we compared them with the genomes of six nonhuman primates assembled by T2T consortium. Out of the 45284 NRSs, 25.3% (11 465) were found in nonhuman primate genomes (Supplementary Table S10). The NRSs overlapping with the human genome were consistent with primate divergence (77), with 8641, 8492, 7717, 5342, 5275 and 4245 NRSs found in the genomes of chimpanzee, bonobo, gorilla, Sumatran orangutan, Bornean orangutan and siamang gibbon, respectively (Supplementary Table S10). In addition, 2049 NRSs were specific to the nonhuman primate genomes, suggesting that these NRSs likely emerged during the process of evolution and divergence. A total of 2354 NRSs were present in all three nonhuman primates, implying that these NRSs originated from a common ancestor (Supplementary Figure S6F).

### Functional annotation of NRSs

Like other genetic variants, NRSs have the potential for functional significance, potentially undergoing transcription (78). To explore this, we initially screened NRSs for the presence of known functional domains using two databases, the NCBI Conserved Domain Database (CDD) (61) and the Pfam database (60). Our analysis revealed that 118 annotated NRSs, featuring recognized functional domains, were associated with 134 genes. Remarkably, 119 (88.8%) of these genes were substantiated through RNA sequencing data or the T2T-CHM13 genome (Supplementary Table S11), underscoring the transcriptional potential and functional significance of these NRSs. Additionally, all 134 genes exhibited homologous counterparts in nonhuman primates, hinting at their origin through gene duplication in evolutionary processes.

To gain deeper insights into how NRSs interact with genes, particularly those relevant to disease, we conducted annotations regarding the insertion sites of NRSs. Among all the NRSs, 20 040 (54.4%) were located within intergenic regions, while 16 813 (45.6%) overlapped with known genes (Figure 2D and Supplementary Table S12). Among those within genic regions, 16 313 NRSs were positioned in gene introns. In addition, 446 NRSs intersected with the exons of 322 protein-coding genes, and 117 NRSs intersected with 96 non-coding genes (Figure 2E). The majority (77.0%) of NRSs intersecting with the exons of protein-coding genes tended to have a low frequency (AF < 0.1) (Supplementary Figure S6G), which is similar to the findings of the previous study (19). Particularly, 84 NRSs intersected with exons of 70 protein-coding genes listed in the Online Mendelian Inheritance in Man (OMIM) catalogue (Supplementary Table S12), suggesting that these NRSs may have a functional impact on disease.

STRs, characterized by the tandem repetition of short DNA sequence motifs (1–6 bp), have been implicated in over 60 distinct phenotypes (79). Triplet repeats, in particular, have recently gained attention for their association with multiple neurodegenerative disorders (80). We identified 210 NRSs containing triplet repeats, of which 102 NRSs intersected with 100 distinct genes, including 28 genes cataloged in the OMIM database (Supplementary Table S13). For instance, we detected a 678-copy CTG repeat expansion in *ATXN8OS*, which was reported to be associated with amyotrophic lateral sclerosis (81); a 429-copy gain of a CGG repeat in *ZNF713*, which was reported to be associated with the folate-sensitive fragile site FRA7A (82); and a 235-copy gain of ACC repeats in *GRIK4*, which contributed to the risk of schizophrenia (83). VNTRs are tandem repeats with motif length ≥7 bp and have been reported to affect diverse human phenotypes through intersecting with protein-coding exons (84). In our investigation, we identified 19 NRSs composed of VNTRs located within the exons of the protein-coding genes, including nine reported in a previous study (Supplementary Table S14) (84). We observed the presence of VNTRs within mucin family genes, such as *MUC2* and *MUC6*. Previous study has demonstrated the association between *MUC6* VNTR expansion and Alzheimer pathologic severity (85). In this study, we detected the expansion of VNTR within *MUC6* with a repeat length of 672 bp (copies ranging from 2.5 to 7.1). Similarly, within *MUC2*, we detected variable expansions with 24-bp repeats (copies ranging from 37.5 to 141.9). Collectively, our datasets provide a resource to investigate the role of tandem repeat expansions in contributing to various phenotypes.

Annotated NRSs could also provide important clues into gene evolution. For instance, we identified a 3.6-kb NRS inserted into the front of a truncated pseudogene *PABPC1P10* (Figure 2F), which is homologous to *PABPC1* that regulates the metabolism of mRNA (86). This observation suggests the existence of a sequence containing the complete pseudogene for *PABPC1* in nonhuman primates before human divergence. This, in turn, raises the possibility that the pseudogene might be redundant or that loss-of-function (LoF) variants were tolerated during human evolution. In contrast, some NRSs expanded after the human divergence. We found a novel NRS consisting of a VNTR with a repeat unit of 24 bp (Figure 2G) located in the second intron of *AFAP1* that was associated with small intestine cancer and open-angle glaucoma (87). Furthermore, this NRS intersected with H3K27Ac, suggesting its potential in regulating gene expression. Notably, we observed either an absence or minimal representation of this VNTR in the nonhuman primate genomes, while diverse human populations displayed a range of 50–270 copies within their genomes. This finding strongly implies that the VNTR in *AFAP1* underwent a substantial expansion after the human divergence from a common great ape ancestor.

### Construction and utility of a graph-based pangenome of human NRSs

The linear reference genome has been found to be insufficient and prone to bias when detecting genetic variations (8). Recent studies have demonstrated the advantages of a graph genome for variant genotyping due to its enhanced sensitivity compared to linear reference-based alignment (88,89). Our graph-based genome was constructed using the current reference genome GRCh38 as the backbone and by incorporating 36853 placed NRSs as the nodes, enabling the creation of alternative paths (Figure 1C and Materials and methods).

To evaluate the impact of the graph pangenome of NRSs on alignment, we mapped publicly available DNA and RNA short-read sequences from diverse populations (62) to GRCh38 and our pangenome. We observed a significantly improvement in the mapping rate for both DNA sequences (from 97.69% to 99.28%, *P* = 0.0059, Wilcoxon signed-rank test) and RNA sequences (from 97.43% to 98.50%, *P* = 0.002, Wilcoxon signed-rank test) (Supplementary Figure S7). This result suggests that the graph pangenome can enhance the detection of novel variants and the quantification of gene expression.

To assess the genotyping accuracy facilitated by the graph genome, we initially compared the genotypes of NRSs that overlapped with INSs of HG002. The sensitivity of the NRS genotypes was 0.94 (Supplementary Table S15), surpassing that of the non-SNPs (0.89 in 1000 Genome + GIAB) reported in a previous study (46), while maintaining a precision of 0.95. To further assess genotype accuracy, we examined the offspring genotypes in five trio datasets (90). The results showed an average Mendelian inheritance abnormality of 2.13%, indicating a low genotyping error rate (Supplementary Table S16). To further assess the genotyping performance at a population scale, we analysed the AF and heterozygosity of the NRSs and examined how many NRSs fit HWE (Supplementary Figure S8). We found that 94.0% of the NRSs showed no significant deviation when testing for HWE, surpassing a previous study on SVs (HWE = 90.7–90.9%) (91). These analyses collectively affirm the reliability of our graph pangenome in genotyping NRSs.

### NRSs affect gene expression

**Figure 3.**
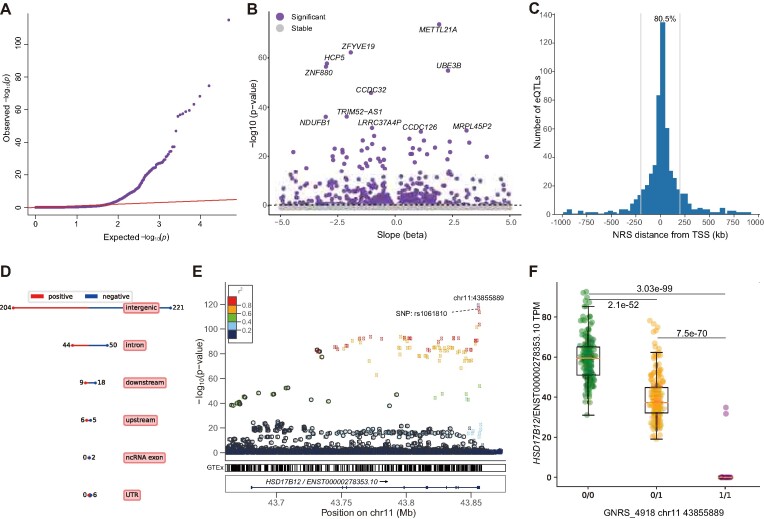
eQTL analysis based on the graph pangenome. (**A**) Quantile-quantile plot of permutation *P* values for all NRS transcript pairs tested. (**B**) Volcano plot of eQTLs and the estimated effect (beta) of the alternative NRS allele on transcript expression. (**C**) Distribution of the distance of significant NRS eQTLs from the TSS of the associated genes. The blue line indicates the position of 200 kb from the TSS. (**D**) Summary of the annotation and impact of eQTL-associated NRSs. ‘positive’ and ‘negative’ indicate the NRS allele increases and decreases the expression of corresponding transcript. (**E**) An example of NRS lead-eQTL for *HSD17B12*. The y-axis represents the significance of the association, with the top eQTL being the highest point. The colours indicate the LD values between the top signal and other variants. (**F**) The median transcripts per million (TPM) for the transcript (ENST00000278353) of *HSD17B12* for individuals containing different NRS alleles. ‘0/0’: homozygous allele same as the reference (*n* = 187); ‘0/1’: heterozygous NRS (*n* = 211); ‘1/1’: homozygous NRS (*n* = 53). Boxes represent the median and quartiles, whiskers extend from the box up to 1.5 times the interquartile range. The *P* values between different alleles were calculated based on a two-tailed t test.

It has been suggested that NRSs may serve as the causal variants for eQTLs, and their longer length makes them more likely to influence the gene expression compared to SNPs (92). In our study, we conducted eQTL analysis using the graph pangenome, and integrated NRS genotypes and RNA-seq data to assess eQTLs in a cohort of 451 samples from the GEUVADIS consortium. PCA based on the graph-based genotyping of NRSs showed that the samples consisted of four EUR ancestry populations and one AFR ancestry population (62), consistent with the PCA result obtained from GEUVADIS consortium based on SNPs (Supplementary Figure S9). Subsequently, we performed the association analysis between transcript expression levels and the graph-based genotypes of NRSs within 1 Mb from the transcription start site (TSS) (93). As a result, we identified a total of 565 NRS-transcript pairs displaying significant expression associations with a FDR <0.05 (Figure 3A and B). Among these pairs, 139 eQTLs were previously reported and 426 (75.4%) were novel discoveries (Supplementary Table S17) (63,93,94).

NRSs that exerted significant effects on gene expression tended to be located proximal to the genes that they regulated, with 80.5% of significant NRS eQTLs occurring within 200 kb of the corresponding TSS (Figure 3C), consistent with the findings of a previous study (93). The NRSs exhibited the capacity to both upregulate (263 NRSs) and downregulate (302 NRSs) the expression of nearby genes (Figure 3D). Significantly, six NRSs located in the 3′UTR of protein-coding genes, along with two NRSs situated in the exon of noncoding genes, exerted a notable impact on the respective genes (Supplementary Table S18). In addition, NRSs may exert various effects on distinct transcripts of the same gene, which may be related to their exact location. Among the NRS-gene pairs, the genes demonstrated significant enrichment in MHC class II receptor activity (GO:0032395) in the GO analysis (odds ratio = 46.6, adjusted *P* = 3.5 × 10^−4^, Fisher's exact test, Supplementary Table S19), suggesting that NRSs might mediate immune diversity by regulating gene expression. To further elucidate the mechanisms by which NRSs influence gene expression, we annotated these NRSs using epigenetic state information of GM12878 lymphoblastoid cells (E116) generated by the Roadmap Epigenetics Consortium (REC) (95). The NRSs exhibited intersections with 12 epigenetic states (Supplementary Table S17) and displayed a strong enrichment in the transcribed state at the 5′ and 3′ end of genes (odds ratio = 17.0, adjusted *P* = 1.6 × 10^−6^, Fisher's exact test, Supplementary Figure S10), as well as the states of TSS, transcription and enhancers. Moreover, we observed a strong depletion of expression associations for NRSs that intersected with the quiescent state that is devoid of important epigenetic marks (odds ratio = 0.3, adjusted *P* = 1.4 × 10^−23^, Fisher's exact test, Supplementary Figure S10).

Furthermore, we found that 15 NRSs (2.7%) exhibited a more significant impact on gene expression than SNPs, with six of them located within the genic region of the affected genes (Supplementary Table S20). For instance, numerous SNPs were found to be significantly associated with the transcript expression of the gene *HSD17B12* (ENST00000278353) which encodes 17 beta-hydroxysteroid dehydrogenase and is associated with long-chain fatty acid metabolism (96). The top SNP signal, rs1061810 (*P* = 2.3 × 10^−117^), was in the 3′UTR of the transcript. Interestingly, we observed that an NRS (GNRS_4918, chr11:43855889, 318 bp) located within the same 3′UTR region exhibited a high LD (*r*^2^ = 0.87) with the SNP rs1061810 and displayed even greater significance (*P* = 5.6 × 10^−120^) (Figure 3E). Additionally, the genotypes of this NRS in the population were in accordance with HWE (*P* = 0.61), indicating accurate genotyping. Furthermore, when examining the median transcripts per million (TPM) for genotypes ‘0/0’, ‘0/1’ and ‘1/1’, we observed values of 59.2, 39.6 and 1.3, respectively (Figure 3F). This finding suggests that NRSs may be associated with gene expression and can serve as eQTL.

### NRSs contribute to the local adaptation of diverse populations

**Figure 4.**
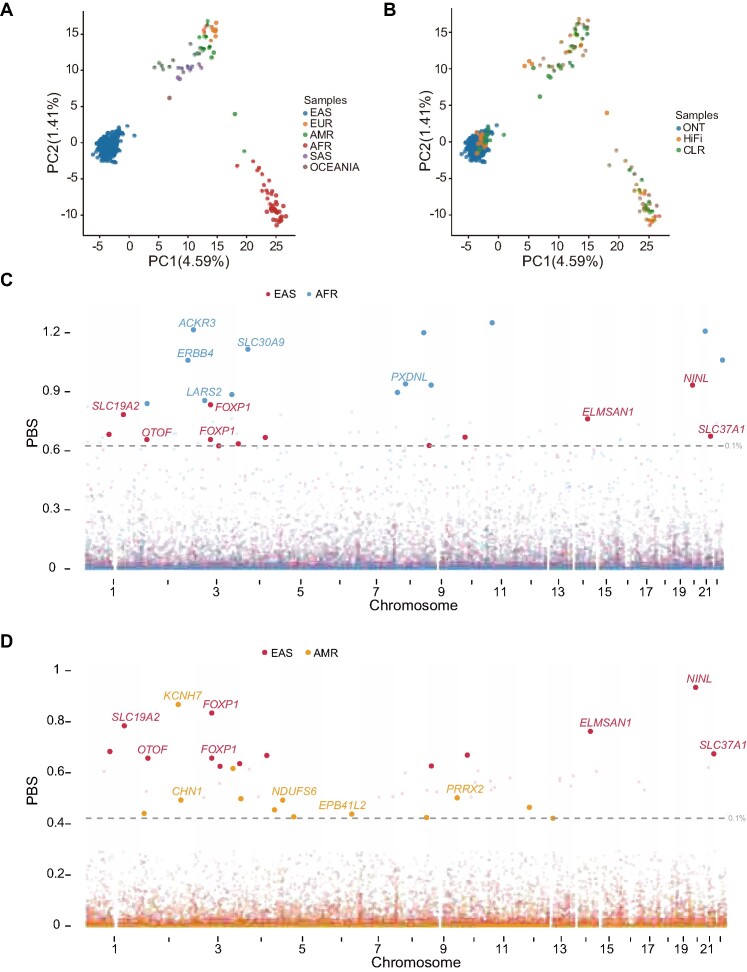
Local adaptation of the NRSs in diverse populations. (**A**) PCA of all the samples across different populations. The values in parentheses indicate the genetic variations explained by the first two PCs. (**B**) PCA of all the samples across different sequencing platforms. (**C**) PBS of NRSs for East Asians and Africans. The grey dotted line represents the top 0.1% (0.62) of the PBS ranked score. (**D**) PBS of NRSs for East Asians and Americans. The grey dotted line represents the top 0.1% (0.42) of the PBS ranked score.

In this study, with the exclusion of three samples with unknown origins and one from Oceania, the remaining samples represented five distinct human populations. The PCA results based on NRS genotypes proficiently separated these samples into their respective population groups, affirming the accuracy of NRS genotypes (Figure 4A). Furthermore, it is important to note that samples from different sequencing platforms within the same population consistently clustered together (Figure 4B), indicating that inter-platform batch effects were minimal and had limited impact on the analysis of population-specific adaptation. To identify NRSs that have undergone population-specific adaptation, we evaluated population differentiation using the population branch statistic (PBS). This analysis leveraged the genotypes of NRSs within EAS, AFR and AMR populations, which collectively represented a large proportion of individuals in our study. We detected 26 unique NRSs with significant PBS scores (top 0.1%) located in or near 25 distinct genes, suggesting their potential as loci associated with population-specific adaptations (Figure 4C, D and Supplementary Tables S21, S22). Remarkably, six of these genes (*KCNH7*, *LUZP2*, *SLC19A2*, *SLC30A9*, *SLC37A1* and *TAF1B*) have been previously documented in the catalogue of human genome adaptation (97). This reinforces the ability of NRSs to unveil signals of local adaptation within human populations.

Some significantly differential NRSs were located within or in close proximity to genes related to metabolism, such as the members of the solute carrier (SLC) superfamily, responsible for transporting extraordinarily diverse solutes across biological membranes (Figure 4C, D and Supplementary Tables S21, S22). For instance, we observed an NRS (GNRS_22864) in the intron of *SLC30A9* encoding a zinc transporter, with AFs of 0.96 and 0.12 in the EAS and AFR populations, respectively. Previous study had suggested that *SLC30A9* underwent natural selection in both EAS and AFR populations, albeit in opposite directions. This divergence is believed to result from local adaptation, influenced by the different zinc state or dietary practices prevalent in these populations (98). Furthermore, we detected a 654-bp NRS (GNRS_19380), located in the intron of *SLC37A1*. This gene plays a role in glucose homeostasis, sugar transport (99), and ion transport that is likely to influence milk mineral composition (100). Previous studies have suggested that genes associated with thiamine metabolisms, including *SLC19A2*, underwent positive selection within the EAS population (101,102). The presence of a 333-bp NRS (GNRS_1744), characterized by a high AF of 0.69 in EASs and a low AF of 0.04 in AFRs, confirms the existence of selection acting upon *SLC19A2* in these populations.

Furthermore, we detected NRSs with potential adaptation signals that may be related to type 2 diabetes (T2D). We found an NRS (GNRS_17568) located within the intron of *ERBB4* (Supplementary Table S21) which was revealed to be associated with T2D and obesity (103).Additionally, an NRS (GNRS_20155) was found to be 9.6 kb upstream of *PIM3* and intersect with H3K4Me1 and transcription factor (TF) clusters (Supplementary Figure S11A), suggesting the potential role of this NRS in regulating gene expression. *PIM3* was reported to be associated with T2D by aggregating genome-wide genotyping data from 32 European-descent GWASs (*P* = 2.0 × 10^−8^) (104). Furthermore, an NRS (GNRS_32802) was found 158 kb upstream of *DMRTA1*. The SNP (rs1575972) near *DMRTA1* was reported to be significantly associated with T2D (*P* = 4.7 × 10^−13^) (105). The NRS was situated in a genomic region flanking three SNPs that were significantly associated with diabetes (*P* = 2.4 × 10^−11^ to 1.8 × 10^−11^) and in high LD (*r*^2^ > 0.8) with the top signal (Supplementary Figure S11B). These findings suggested that the adaptation of the NRSs related to these diabetes-related genes might contribute to the difference in diabetes incidence in different populations.

### Potential function of NRSs to phenotypic variation

**Figure 5.**
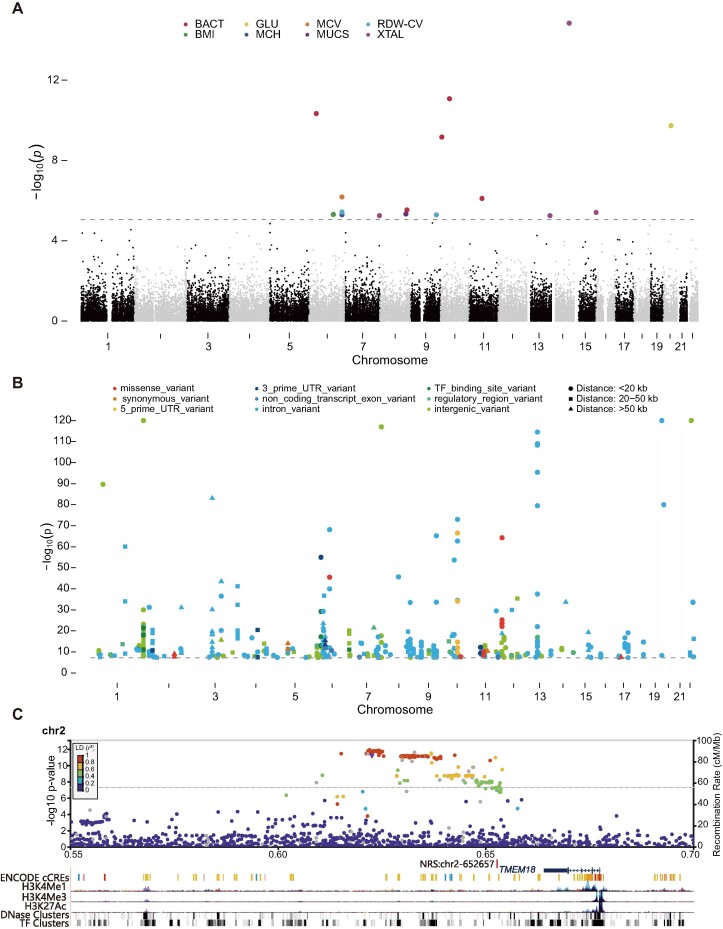
NRSs. significantly associated with phenotypes. (**A**) Manhattan plots show NRSs plotted on the x-axis according to their position on each chromosome against, on the y-axis (shown as − log_10_*P* value), the association with clinical phenotypes. The grey dotted line indicates the significance threshold (*P* = 8.9 × 10^−6^ through Bonferroni correction). BACT: urinary bacteria, GLU: blood glucose, MCV: mean corpuscular volume, RDW-CV: red cell volume distribution width- coefficient of variation, BMI: body mass index, MCH: mean corpuscular haemoglobin, MUCS: mucus, XTAL: urinary crystal. (**B**) Manhattan plot for the phenotype-associated SNPs that are in strong LD (*r*^2^ > 0.8) with NRSs. Different colours demonstrate different gene features in the GWAS catalogue, and the shapes indicate the distances between the SNP and the NRS. (**C**) Regional SNP association plots with the NRS (red vertical line) around *TMEM18* shown in high LD (*r*^2^ > 0.8) with the top signal of BMI.

We next conducted a GWAS on NRS genotypes and clinical traits using our constructed graph pangenome. Our study included 327 samples with 68 traits obtained during health check-ups (19). We employed an additive genetic model with relevant covariates for the quantitative traits using the 5643 NRSs with a MAF greater than 0.05. The genomic inflation factor (λ_GC_) exhibited values ranging from 1.00 to 1.05, with an average of 1.01, indicating very low inflation. Finally, we identified 14 NRSs significantly associated with eight phenotypes (*P* < 8.9 × 10^−6^, the Bonferroni-corrected significance threshold, Figure 5A and Supplementary Table S23). For example, one of these NRSs, GNRS_28218, was significantly associated with mean corpuscular haemoglobin (MCH), mean corpuscular volume (MCV) and red cell volume distribution width-coefficient of variation (RDW-CV), which serve as indicators for assessing anaemia. GNRS_28218 resides in the intron of the interaction protein for cytohesin exchange factors 1 (*IPCEF1*). A previous GWAS of chronic lymphocytic leukaemia (CLL) identified a susceptibility locus mapping to *IPCEF1* (rs2236256, *P* = 2 × 10^−10^) (106). CLL is frequently complicated by cytopaenias, either due to bone marrow infiltration or autoimmunity, and results in autoimmune haemolytic anaemia (AIHA) (107), suggesting the potential involvement of *IPCEF1* in anaemia. The GO annotation shows that *IPCEF1* is related to peroxidase activity (GO:0004601) and oxygen carrier activity (GO:0005344). The GenomeRNAi database reveals that RNA interference with *IPCEF1* in human leads to decreased endocytosis of transferrin (108), which has an impact on iron incorporation by erythroblasts. Collectively, this evidence suggested that GNRS_28218, associated with *IPCEF1*, is likely to have a functional role in anaemia.

To further explore the potential functions of NRSs in phenotypes, we identified NRSs that were in LD with SNPs associated with phenotypes in the GWAS catalogue (72). Using a window size of 100 kb, we observed strong LD (*r*^2^ > 0.8) between 154 NRSs and 258 phenotype-associated SNPs at genome-wide significance (*P* = 5 × 10^−8^) reported in the GWAS catalogue (Figure 5B, Supplementary Table S24 and Materials and methods). Notably, an NRS, GNRS_15339 on chromosome 2 (2p25.3) was located a mere 266 bp away from *cis-*regulatory elements and exhibited strong LD with 15 unique SNPs. These SNPs were found in regulatory or intergenic regions near *TMEM18* in 15 GWASs and were significantly associated with body mass index (BMI) (Figure 5C). Further analysis revealed that seven additional NRSs (GNRS_21065, 27160, 30214, 30546, 33543, 12548 and 12873) were in strong LD with SNPs that were also significantly associated with BMI. All these SNPs were positioned in intergenic, regulatory or intron regions, except for a missense variant (rs17826219, *P* = 3 × 10^−8^) in *ATAD5*. However, these eight NRSs were not significantly associated with BMI (*P* = 0.0046 for GNRS_30214) in our GWAS, suggesting that a larger sample size is needed in future study. Interestingly, of the 258 phenotype-associated SNPs in strong LD with NRSs, only 11 (4.3%) SNPs were found in the coding region. Seven of these SNPs were missense variants and four were synonymous variants. Furthermore, 25 (16.2%) NRSs with high LD with these phenotype-associated SNPs were validated to significantly regulate the gene expression through eQTL analysis (Supplementary Table S24). This suggests that these NRSs may possess the potential to affect the phenotypes by regulating the expression of nearby genes. However, further functional studies are needed to validate any causal roles for these NRSs in phenotype regulation.

## Discussion

Numerous genetic sequences are missing from the current reference genome GRCh38, particularly those originating from diverse human populations. To comprehensively unravel the genetic variations in human populations, it is crucial to construct a human pangenome reference that incorporates population-level diversity (3). To better characterize human NRSs and decipher their functional significance, we conducted an in-depth study involving 539 human genomes utilizing LRS technology. Our method of identifying NRSs from the assemblies of LRS data offered a distinct advantage, primarily because majority substantial portion of NRSs comprised of low-complexity sequences. In this study, we observed that the average length of assembled contigs for each individual reached 17.0 Mb, which was slightly longer than the length reported in previous long-read assemblies (from 12.8 to 16.0 Mb) (3), and significantly surpassed the lengths of contigs obtained from short reads (from 0.2 to 2.5 Mb). More importantly, our LRS-based datasets included NRSs originating from multiple human populations, resulting in a more comprehensive pangenome than previous studies mainly focusing on a single population or small sample sizes (4,11,51). As a result of this extensive effort, we identified a substantial number of novel NRSs, accounting for 29.7% (13441) of all discovered NRSs. Moreover, we successfully annotated 118 NRSs with known functional domains, linking them to 134 genes, and impressively, 88.8% of these were validated by the RNA dataset or T2T-CHM13. Intriguingly, we located 446 NRSs within the exons of 322 protein-coding genes, indicating their potential to disrupt gene function. Furthermore, our comprehensive analysis of the distribution of NRSs across the human genome and within the human populations, as well as in nonhuman primates, provided valuable insights into the evolution of NRSs. This extended our understanding of the prevalence of human NRSs and their functional impact on evolution, phenotypes and diseases.

Our study demonstrated the efficacy of using a graph pangenome approach for analyzing NRSs, leading to an enhanced sequence mapping rates. This method has the potential to discover more variants and thus provides a more comprehensive understanding of genetic variations related to phenotypes and diseases. Importantly, the graph pangenome facilitates the genotyping of NRSs, presenting an opportunity to discover novel associations between NRSs and phenotypes. Our result indicated additional eQTLs beyond SNPs, shedding light on the regulation of gene expression by NRSs. Furthermore, we found strong LD between genotyped NRSs and phenotype-associated SNPs in the GWAS catalogue. Since most of these SNPs (95.7%) were in noncoding regions of genes, a substantial proportion are unlikely to be causal variants that may hold keys to missing heritability.

Currently, a substantial portion of NRSs cannot be anchored to the reference genome, mainly due to the inherent challenges associated with assembling larger repetitive regions. Recent advancements, such as the integrating of ONT ultralong and PacBio HiFi reads, offer promising prospects for the accurate detection of NRSs within repetitive regions, including SDs and centromeric regions. This is exemplified by the successful T2T assembly on the haploid CHM13 genome which added nearly 200 Mb sequence compared to GRCh38 and revealed hundreds of thousands of previously unresolved variants (2,109). Incorporating deep sequencing data from more multiple platforms, such as Bionano optical maps and Hi-C Illumina short-read sequencing, as demonstrated by the study conducted by Liao *et al.*, resulted in the achievement of 47 state-of-the-art phased, diploid assemblies within HPRC (16). To further enhance the assembly quality of the genomes and accurately extract NRSs, particularly within low-complexity regions such as SDs, future research endeavors will necessitate larger volumes of deep sequencing data generated from diverse platforms. Considering the increased completeness of the T2T-CHM13 genome, it has the potential to enhance the detection of genetic variants (109). To evaluate the performance of identifying NRSs against T2T-CHM13, we randomly selected 23 samples from five populations. The average number of placed NRSs decreased by 109 after using T2T-CHM13 (from 8577 using GRCh38 to 8468 using T2T-CHM13) (Supplementary Figure S12). While the average number of unplaced NRSs exhibited a substantial reduction of 735 (from 834 using GRCh38 to 99 using T2T-CHM13). This implies the majority of technical NRSs were unplaced. Considering that our study primarily focused on placed NRSs, the small reduction (1.3%) in the number of placed NRSs suggests that replacing the reference genome GRCh38 with T2T-CHM13 has marginal impact on current results of the downstream analyses.

For a more comprehensive analysis of local adaptation and GWAS, increased sample sizes encompassing a broader range of phenotypes are imperative, with special emphasis on the AFR population, known for its heightened genetic diversity compared to other populations. As sample sizes expand and genetic diversity increases, the number of graph nodes housing multiple variants will correspondingly rise, leading to improved alignment precision and genotyping accuracy. It should be noted that, in this study, we included a substantial number of EAS samples in our pangenome, comprising almost 83% of the total samples. While this ensured adequate Asian representation, it introduced bias toward Asian-specific NRSs. The future work could incorporate more samples which represent broader global ethnic diversity.

In summary, our efforts to identify and characterize human NRSs elucidated the evolutionary and functional significance of these NRSs, while producing a valuable resource for human genomic research. The resulting graph-based pangenome also enables more robust analyses of eQTLs, population-level local adaptation, and genotype-phenotype association. This represents a critical step towards realizing a comprehensive global human pangenome. Overall, this work highlighted the importance of incorporating NRSs into the human reference genome to fully capture genetic diversity and understand its impacts on biology and disease.

## Supplementary Material

gkae086_Supplemental_Files

## Data Availability

The sequencing data for all 539 individuals in this study are publicly available. Detail information about these datasets is provided in Supplementary Table S1. The sequences and genotypes of the nonredundant NRSs are publicly accessible through the National Genomics Data Center (NGDC), China National Center for Bioinformation (CNCB), with the accession number GVM000672 (https://ngdc.cncb.ac.cn/gvm/getProjectDetail?project=GVM000672). Additionally, the data are available in Zenodo at https://doi.org/10.5281/zenodo.10554485. They are also available in GitHub at https://github.com/xie-lab/GNRS/tree/main/NRS. The codes of pipeline GraphNRS in this study are publicly available via GitHub repository (https://github.com/xie-lab/GNRS).

## References

[1] Lappalainen T., Scott A.J., Brandt M., Hall I.M. Genomic analysis in the age of Human genome sequencing. Cell. 2019; 177:70–84. 30901550 10.1016/j.cell.2019.02.032 PMC6532068

[2] Nurk S., Koren S., Rhie A., Rautiainen M., Bzikadze A.V., Mikheenko A., Vollger M.R., Altemose N., Uralsky L., Gershman A. et al. The complete sequence of a human genome. Science. 2022; 376:44–53. 35357919 10.1126/science.abj6987 PMC9186530

[3] Sherman R.M., Salzberg S.L. Pan-genomics in the human genome era. Nat. Rev. Genet. 2020; 21:243–254. 32034321 10.1038/s41576-020-0210-7 PMC7752153

[4] Kehr B., Helgadottir A., Melsted P., Jonsson H., Helgason H., Jonasdottir A., Jonasdottir A., Sigurdsson A., Gylfason A., Halldorsson G.H. et al. Diversity in non-repetitive human sequences not found in the reference genome. Nat. Genet. 2017; 49:588–593. 28250455 10.1038/ng.3801

[5] Wong K.H.Y., Ma W., Wei C.Y., Yeh E.C., Lin W.J., Wang E.H.F., Su J.P., Hsieh F.J., Kao H.J., Chen H.H. et al. Towards a reference genome that captures global genetic diversity. Nat. Commun. 2020; 11:5482. 33127893 10.1038/s41467-020-19311-w PMC7599213

[6] Wong K.H.Y., Levy-Sakin M., Kwok P.Y. De novo human genome assemblies reveal spectrum of alternative haplotypes in diverse populations. Nat. Commun. 2018; 9:3040. 30072691 10.1038/s41467-018-05513-w PMC6072799

[7] Duan Z., Qiao Y., Lu J., Lu H., Zhang W., Yan F., Sun C., Hu Z., Zhang Z., Li G. et al. HUPAN: a pan-genome analysis pipeline for human genomes. Genome Biol. 2019; 20:149. 31366358 10.1186/s13059-019-1751-y PMC6670167

[8] Lee Y.G., Lee J.Y., Kim J., Kim Y.J. Insertion variants missing in the human reference genome are widespread among human populations. BMC Biol. 2020; 18:167. 33187521 10.1186/s12915-020-00894-1 PMC7666470

[9] Chu C., Borges-Monroy R., Viswanadham V.V., Lee S., Li H., Lee E.A., Park P.J. Comprehensive identification of transposable element insertions using multiple sequencing technologies. Nat. Commun. 2021; 12:3836. 34158502 10.1038/s41467-021-24041-8 PMC8219666

[10] Meleshko D., Yang R., Marks P., Williams S., Hajirasouliha I. Efficient detection and assembly of non-reference DNA sequences with synthetic long reads. Nucleic Acids Res. 2022; 50:e108. 35924489 10.1093/nar/gkac653 PMC9561269

[11] Li Q., Tian S., Yan B., Liu C.M., Lam T.W., Li R., Luo R. Building a Chinese pan-genome of 486 individuals. Commun. Biol. 2021; 4:1016. 34462542 10.1038/s42003-021-02556-6 PMC8405635

[12] Jain M., Koren S., Miga K.H., Quick J., Rand A.C., Sasani T.A., Tyson J.R., Beggs A.D., Dilthey A.T., Fiddes I.T. et al. Nanopore sequencing and assembly of a human genome with ultra-long reads. Nat. Biotechnol. 2018; 36:338–345. 29431738 10.1038/nbt.4060 PMC5889714

[13] Shi L., Guo Y., Dong C., Huddleston J., Yang H., Han X., Fu A., Li Q., Li N., Gong S. et al. Long-read sequencing and de novo assembly of a Chinese genome. Nat. Commun. 2016; 7:12065. 27356984 10.1038/ncomms12065 PMC4931320

[14] Ameur A., Che H., Martin M., Bunikis I., Dahlberg J., Hoijer I., Haggqvist S., Vezzi F., Nordlund J., Olason P. et al. De Novo assembly of two Swedish genomes reveals missing segments from the Human GRCh38 reference and improves variant calling of population-scale sequencing data. Genes (Basel). 2018; 9:486. 30304863 10.3390/genes9100486 PMC6210158

[15] Wang T., Antonacci-Fulton L., Howe K., Lawson H.A., Lucas J.K., Phillippy A.M., Popejoy A.B., Asri M., Carson C., Chaisson M.J.P. et al. The Human Pangenome Project: a global resource to map genomic diversity. Nature. 2022; 604:437–446. 35444317 10.1038/s41586-022-04601-8 PMC9402379

[16] Liao W.-W., Asri M., Ebler J., Doerr D., Haukness M., Hickey G., Lu S., Lucas J.K., Monlong J., Abel H.J. et al. A draft human pangenome reference. Nature. 2023; 617:312–324. 37165242 10.1038/s41586-023-05896-x PMC10172123

[17] Gao Y., Yang X., Chen H., Tan X., Yang Z., Deng L., Wang B., Kong S., Li S., Cui Y. et al. A pangenome reference of 36 Chinese populations. Nature. 2023; 619:112–121. 37316654 10.1038/s41586-023-06173-7 PMC10322713

[18] Uddin M., Nassir N., Almarri M., Kumail M., Mohamed N., Balan B., Hanif S., AlObathani M., Jamalalail B., Elsokary H. et al. A draft Arab pangenome reference. Res. Square. 2023; 3:3490341. 10.1038/s41467-025-61645-w PMC12290100 40707445

[19] Wu Z., Jiang Z., Li T., Xie C., Zhao L., Yang J., Ouyang S., Liu Y., Li T., Xie Z. Structural variants in the Chinese population and their impact on phenotypes, diseases and population adaptation. Nat. Commun. 2021; 12:6501. 34764282 10.1038/s41467-021-26856-x PMC8586011

[20] Ebert P., Audano P.A., Zhu Q., Rodriguez-Martin B., Porubsky D., Bonder M.J., Sulovari A., Ebler J., Zhou W., Serra Mari R. et al. Haplotype-resolved diverse human genomes and integrated analysis of structural variation. Science. 2021; 372:eabf7117. 33632895 10.1126/science.abf7117 PMC8026704

[21] Quan C., Li Y., Liu X., Wang Y., Ping J., Lu Y., Zhou G. Characterization of structural variation in Tibetans reveals new evidence of high-altitude adaptation and introgression. Genome Biol. 2021; 22:159. 34034800 10.1186/s13059-021-02382-3 PMC8146648

[22] Ruan J., Li H. Fast and accurate long-read assembly with wtdbg2. Nat. Methods. 2020; 17:155–158. 31819265 10.1038/s41592-019-0669-3 PMC7004874

[23] Shafin K., Pesout T., Lorig-Roach R., Haukness M., Olsen H.E., Bosworth C., Armstrong J., Tigyi K., Maurer N., Koren S. et al. Nanopore sequencing and the Shasta toolkit enable efficient de novo assembly of eleven human genomes. Nat. Biotechnol. 2020; 38:1044–1053. 32686750 10.1038/s41587-020-0503-6 PMC7483855

[24] Cheng H., Concepcion G.T., Feng X., Zhang H., Li H. Haplotype-resolved de novo assembly using phased assembly graphs with hifiasm. Nat. Methods. 2021; 18:170–175. 33526886 10.1038/s41592-020-01056-5 PMC7961889

[25] Gurevich A., Saveliev V., Vyahhi N., Tesler G. QUAST: quality assessment tool for genome assemblies. Bioinformatics. 2013; 29:1072–1075. 23422339 10.1093/bioinformatics/btt086 PMC3624806

[26] Chen Y., Zhang Y., Wang A.Y., Gao M., Chong Z. Accurate long-read de novo assembly evaluation with Inspector. Genome Biol. 2021; 22:312. 34775997 10.1186/s13059-021-02527-4 PMC8590762

[27] Shumate A., Zimin A.V., Sherman R.M., Puiu D., Wagner J.M., Olson N.D., Pertea M., Salit M.L., Zook J.M., Salzberg S.L. Assembly and annotation of an Ashkenazi human reference genome. Genome Biol. 2020; 21:129. 32487205 10.1186/s13059-020-02047-7 PMC7265644

[28] Pedersen B.S., Quinlan A.R. Mosdepth: quick coverage calculation for genomes and exomes. Bioinformatics. 2018; 34:867–868. 29096012 10.1093/bioinformatics/btx699 PMC6030888

[29] Hayden K.E., Strome E.D., Merrett S.L., Lee H.R., Rudd M.K., Willard H.F. Sequences associated with centromere competency in the human genome. Mol. Cell. Biol. 2013; 33:763–772. 23230266 10.1128/MCB.01198-12 PMC3571344

[30] Altemose N., Miga K.H., Maggioni M., Willard H.F. Genomic characterization of large heterochromatic gaps in the human genome assembly. PLoS Comput. Biol. 2014; 10:e1003628. 24831296 10.1371/journal.pcbi.1003628 PMC4022460

[31] Li H. Identifying centromeric satellites with dna-brnn. Bioinformatics. 2019; 35:4408–4410. 30989183 10.1093/bioinformatics/btz264 PMC6821349

[32] Manni M., Zdobnov E. Microbial contaminants cataloged as novel human sequences in recent human pan-genomes. 2020; bioRxiv doi:18 March 2020, preprint: not peer reviewed 10.1101/2020.03.16.994376.

[33] Benson G. Tandem repeats finder a program to analyze DNA sequences. Nucleic Acids Res. 1999; 27:573–580. 9862982 10.1093/nar/27.2.573 PMC148217

[34] Hubley R., Finn R.D., Clements J., Eddy S.R., Jones T.A., Bao W., Smit A.F., Wheeler T.J. The Dfam database of repetitive DNA families. Nucleic Acids Res. 2016; 44:D81–D89. 26612867 10.1093/nar/gkv1272 PMC4702899

[35] Bao W., Kojima K.K., Kohany O. Repbase Update, a database of repetitive elements in eukaryotic genomes. Mob. DNA. 2015; 6:11. 26045719 10.1186/s13100-015-0041-9 PMC4455052

[36] Menzel P., Ng K.L., Krogh A. Fast and sensitive taxonomic classification for metagenomics with Kaiju. Nat. Commun. 2016; 7:11257. 27071849 10.1038/ncomms11257 PMC4833860

[37] Buchfink B., Xie C., Huson D.H. Fast and sensitive protein alignment using DIAMOND. Nat. Methods. 2015; 12:59–60. 25402007 10.1038/nmeth.3176

[38] Camacho C., Coulouris G., Avagyan V., Ma N., Papadopoulos J., Bealer K., Madden T.L. BLAST+: architecture and applications. BMC Bioinf. 2009; 10:421. 10.1186/1471-2105-10-421 PMC2803857 20003500

[39] Xiao C.L., Chen Y., Xie S.Q., Chen K.N., Wang Y., Han Y., Luo F., Xie Z. MECAT: fast mapping, error correction, and de novo assembly for single-molecule sequencing reads. Nat. Methods. 2017; 14:1072–1074. 28945707 10.1038/nmeth.4432

[40] Abyzov A., Gerstein M. AGE: defining breakpoints of genomic structural variants at single-nucleotide resolution, through optimal alignments with gap excision. Bioinformatics. 2011; 27:595–603. 21233167 10.1093/bioinformatics/btq713 PMC3042181

[41] Hao Z., Lv D., Ge Y., Shi J., Weijers D., Yu G., Chen J. RIdeogram: drawing SVG graphics to visualize and map genome-wide data on the idiograms. PeerJ Computer Science. 2020; 6:e251. 10.7717/peerj-cs.251 PMC7924719 33816903

[42] Li H., Alkan C. New strategies to improve minimap2 alignment accuracy. Bioinformatics. 2021; 37:4572–4574. 34623391 10.1093/bioinformatics/btab705 PMC8652018

[43] Kirsche M., Prabhu G., Sherman R., Ni B., Battle A., Aganezov S., Schatz M.C. Jasmine and Iris: population-scale structural variant comparison and analysis. Nat. Methods. 2023; 20:408–417. 36658279 10.1038/s41592-022-01753-3 PMC10006329

[44] Lassmann T., Sonnhammer E.L. Kalign–an accurate and fast multiple sequence alignment algorithm. BMC Bioinf. 2005; 6:298. 10.1186/1471-2105-6-298 PMC1325270 16343337

[45] Hickey G., Heller D., Monlong J., Sibbesen J.A., Siren J., Eizenga J., Dawson E.T., Garrison E., Novak A.M., Paten B. Genotyping structural variants in pangenome graphs using the vg toolkit. Genome Biol. 2020; 21:35. 32051000 10.1186/s13059-020-1941-7 PMC7017486

[46] Rautiainen M., Marschall T. GraphAligner: rapid and versatile sequence-to-graph alignment. Genome Biol. 2019; 21:253. 10.1186/s13059-020-02157-2 PMC7513500 32972461

[47] Seo J.S., Rhie A., Kim J., Lee S., Sohn M.H., Kim C.U., Hastie A., Cao H., Yun J.Y., Kim J. et al. De novo assembly and phasing of a Korean human genome. Nature. 2016; 538:243–247. 27706134 10.1038/nature20098

[48] Cho Y.S., Kim H., Kim H.M., Jho S., Jun J., Lee Y.J., Chae K.S., Kim C.G., Kim S., Eriksson A. et al. An ethnically relevant consensus Korean reference genome is a step towards personal reference genomes. Nat. Commun. 2016; 7:13637. 27882922 10.1038/ncomms13637 PMC5123046

[49] Levy S., Sutton G., Ng P.C., Feuk L., Halpern A.L., Walenz B.P., Axelrod N., Huang J., Kirkness E.F., Denisov G. et al. The diploid genome sequence of an individual Human. PLoS Biol. 2007; 5:e254. 17803354 10.1371/journal.pbio.0050254 PMC1964779

[50] Steinberg K.M., Lindsay T.G., Schneider V.A., Chaisson M.J.P., Tomlinson C., Huddleston J., Minx P., Kremitzki M., Albrecht D., Magrini V. et al. High-quality assembly of an individual of Yoruban descent. 2016; bioRxiv doi:02 August 2016, preprint: not peer reviewed 10.1101/067447.

[51] Sherman R.M., Forman J., Antonescu V., Puiu D., Daya M., Rafaels N., Boorgula M.P., Chavan S., Vergara C., Ortega V.E. et al. Assembly of a pan-genome from deep sequencing of 910 humans of African descent. Nat. Genet. 2019; 51:30–35. 30455414 10.1038/s41588-018-0273-y PMC6309586

[52] Eisfeldt J., Martensson G., Ameur A., Nilsson D., Lindstrand A. Discovery of novel sequences in 1,000 Swedish genomes. Mol. Biol. Evol. 2019; 37:18–30. 10.1093/molbev/msz176 PMC6984370 31560401

[53] Almarri M.A., Bergstrom A., Prado-Martinez J., Yang F., Fu B., Dunham A.S., Chen Y., Hurles M.E., Tyler-Smith C., Xue Y. Population structure, stratification, and introgression of Human structural variation. Cell. 2020; 182:189–199. 32531199 10.1016/j.cell.2020.05.024 PMC7369638

[54] Beyter D., Ingimundardottir H., Oddsson A., Eggertsson H.P., Bjornsson E., Jonsson H., Atlason B.A., Kristmundsdottir S., Mehringer S., Hardarson M.T. et al. Long-read sequencing of 3,622 Icelanders provides insight into the role of structural variants in human diseases and other traits. Nat. Genet. 2021; 53:779–786. 33972781 10.1038/s41588-021-00865-4

[55] Li W., Godzik A. Cd-hit: a fast program for clustering and comparing large sets of protein or nucleotide sequences. Bioinformatics. 2006; 22:1658–1659. 16731699 10.1093/bioinformatics/btl158

[56] Yandell C.H.M. MAKER2 an annotation pipeline and genome-database management tool for second-generation genome projects. BMC Bioinf. 2011; 12:491. 10.1186/1471-2105-12-491 PMC3280279 22192575

[57] Korf I. Gene finding in novel genomes. BMC Bioinf. 2004; 5:59. 10.1186/1471-2105-5-59 PMC421630 15144565

[58] Stanke M., Keller O., Gunduz I., Hayes A., Waack S., Morgenstern B. AUGUSTUS: ab initio prediction of alternative transcripts. Nucleic Acids Res. 2006; 34:W435–W439. 16845043 10.1093/nar/gkl200 PMC1538822

[59] Campbell M.S., Holt C., Moore B., Yandell M. Genome annotation and curation using MARKR and MARKR-P. Current Protoc. Bioinform. 2014; 48:4.11.1–4.11.39. 10.1002/0471250953.bi0411s48 PMC4286374 25501943

[60] Finn R.D., Coggill P., Eberhardt R.Y., Eddy S.R., Mistry J., Mitchell A.L., Potter S.C., Punta M., Qureshi M., Sangrador-Vegas A. et al. The Pfam protein families database: towards a more sustainable future. Nucleic Acids Res. 2016; 44:D279–D285. 26673716 10.1093/nar/gkv1344 PMC4702930

[61] Marchler-Bauer A., Bo Y., Han L., He J., Lanczycki C.J., Lu S., Chitsaz F., Derbyshire M.K., Geer R.C., Gonzales N.R. et al. CDD/SPARCLE: functional classification of proteins via subfamily domain architectures. Nucleic Acids Res. 2017; 45:D200–D203. 27899674 10.1093/nar/gkw1129 PMC5210587

[62] Lappalainen T., Sammeth M., Friedlander M.R., Hoen P.A., Monlong J., Rivas M.A., Gonzalez-Porta M., Kurbatova N., Griebel T., Ferreira P.G. et al. Transcriptome and genome sequencing uncovers functional variation in humans. Nature. 2013; 501:506–511. 24037378 10.1038/nature12531 PMC3918453

[63] Audano P.A., Sulovari A., Graves-Lindsay T.A., Cantsilieris S., Sorensen M., Welch A.E., Dougherty M.L., Nelson B.J., Shah A., Dutcher S.K. et al. Characterizing the major structural variant alleles of the Human genome. Cell. 2019; 176:663–675. 30661756 10.1016/j.cell.2018.12.019 PMC6438697

[64] Sibbesen J.A., Eizenga J.M., Novak A.M., Siren J., Chang X., Garrison E., Paten B. Haplotype-aware pantranscriptome analyses using spliced pangenome graphs. Nat. Methods. 2023; 20:239–247. 36646895 10.1038/s41592-022-01731-9

[65] Ongen H., Buil A., Brown A.A., Dermitzakis E.T., Delaneau O. Fast and efficient QTL mapper for thousands of molecular phenotypes. Bioinformatics. 2016; 32:1479–1485. 26708335 10.1093/bioinformatics/btv722 PMC4866519

[66] McLaren W., Gil L., Hunt S.E., Riat H.S., Ritchie G.R.S., Thormann A., Flicek P., Cunningham F. The Ensembl variant effect predictor. Genome Biol. 2016; 17:122. 27268795 10.1186/s13059-016-0974-4 PMC4893825

[67] Price A.L., Patterson N.J., Plenge R.M., Weinblatt M.E., Shadick N.A., Reich D Principal components analysis corrects for stratification in genome-wide association studies. Nat. Genet. 2006; 38:904–909. 16862161 10.1038/ng1847

[68] Hämälä T., Savolainen O., Purugganan M. Genomic patterns of local adaptation under gene flow in Arabidopsis lyrata. Mol. Biol. Evol. 2019; 36:2557–2571. 31236594 10.1093/molbev/msz149

[69] Purcell S., Neale B., Todd-Brown K., Thomas L., Ferreira M.A., Bender D., Maller J., Sklar P., de Bakker P.I., Daly M.J. et al. PLINK: a tool set for whole-genome association and population-based linkage analyses. Am. J. Hum. Genet. 2007; 81:559–575. 17701901 10.1086/519795 PMC1950838

[70] Jeon S., Bhak Y., Choi Y., Jeon Y., Kim S., Jang J., Jang J., Blazyte A., Kim C., Kim Y. et al. Korean Genome Project: 1094 Korean personal genomes with clinical information. Sci. Adv. 2020; 6:eaaz7835. 32766443 10.1126/sciadv.aaz7835 PMC7385432

[71] Edge P., Bansal V. Longshot enables accurate variant calling in diploid genomes from single-molecule long read sequencing. Nat. Commun. 2019; 10:4660. 31604920 10.1038/s41467-019-12493-y PMC6788989

[72] Buniello A., MacArthur J.A.L., Cerezo M., Harris L.W., Hayhurst J., Malangone C., McMahon A., Morales J., Mountjoy E., Sollis E. et al. The NHGRI-EBI GWAS Catalog of published genome-wide association studies, targeted arrays and summary statistics 2019. Nucleic Acids Res. 2019; 47:D1005–D1012. 30445434 10.1093/nar/gky1120 PMC6323933

[73] Chen S., Krusche P., Dolzhenko E., Sherman R.M., Petrovski R., Schlesinger F., Kirsche M., Bentley D.R., Schatz M.C., Sedlazeck F.J. et al. Paragraph: a graph-based structural variant genotyper for short-read sequence data. Genome Biol. 2019; 20:291. 31856913 10.1186/s13059-019-1909-7 PMC6921448

[74] Kuleshov M.V., Jones M.R., Rouillard A.D., Fernandez N.F., Duan Q., Wang Z., Koplev S., Jenkins S.L., Jagodnik K.M., Lachmann A. et al. Enrichr: a comprehensive gene set enrichment analysis web server 2016 update. Nucleic Acids Res. 2016; 44:W90–W97. 27141961 10.1093/nar/gkw377 PMC4987924

[75] Du Z., Ma L., Qu H., Chen W., Zhang B., Lu X., Zhai W., Sheng X., Sun Y., Li W. et al. Whole genome analyses of Chinese population and de novo assembly of A Northern Han genome. Genomics Proteomics Bioinformatics. 2019; 17:229–247. 31494266 10.1016/j.gpb.2019.07.002 PMC6818495

[76] Lin Y.L., Gokcumen O. Fine-scale characterization of genomic structural variation in the Human genome reveals adaptive and biomedically relevant hotspots. Genome Biol. Evol. 2019; 11:1136–1151. 30887040 10.1093/gbe/evz058 PMC6475128

[77] Heijmans C.M.C., de Groot N.G., Bontrop R.E. Comparative genetics of the major histocompatibility complex in humans and nonhuman primates. Int. J. Immunogenet. 2020; 47:243–260. 32358905 10.1111/iji.12490

[78] Li R., Tian X., Yang P., Fan Y., Li M., Zheng H., Wang X., Jiang Y. Recovery of non-reference sequences missing from the human reference genome. Bmc Genomics [Electronic Resource]. 2019; 20:746. 31619167 10.1186/s12864-019-6107-1 PMC6796347

[79] Gall-Duncan T., Sato N., Yuen R.K.C., Pearson C.E. Advancing genomic technologies and clinical awareness accelerates discovery of disease-associated tandem repeat sequences. Genome Res. 2022; 32:1–27. 34965938 10.1101/gr.269530.120 PMC8744678

[80] Zhou Z.D., Jankovic J., Ashizawa T., Tan E.K. Neurodegenerative diseases associated with non-coding CGG tandem repeat expansions. Nat. Rev. Neurol. 2022; 18:145–157. 35022573 10.1038/s41582-021-00612-7

[81] Hirano M., Samukawa M., Isono C., Saigoh K., Nakamura Y., Kusunoki S. Noncoding repeat expansions for ALS in Japan are associated with the ATXN8OS gene. Neurol. Genet. 2018; 4:e252. 30109267 10.1212/NXG.0000000000000252 PMC6089696

[82] Metsu S., Rainger J.K., Debacker K., Bernhard B., Rooms L., Grafodatskaya D., Weksberg R., Fombonne E., Taylor M.S., Scherer S.W. et al. A CGG-repeat expansion mutation in ZNF713 causes FRA7A: association with autistic spectrum disorder in two families. Hum. Mutat. 2014; 35:1295–1300. 25196122 10.1002/humu.22683

[83] Mojarad B.A., Engchuan W., Trost B., Backstrom I., Yin Y., Thiruvahindrapuram B., Pallotto L., Mitina A., Khan M., Pellecchia G. et al. Genome-wide tandem repeat expansions contribute to schizophrenia risk. Mol. Psychiatry. 2022; 27:3692–3698. 35546631 10.1038/s41380-022-01575-x PMC9708556

[84] Mukamel R.E., Handsaker R.E., Sherman M.A., Barton A.R., Zheng Y., McCarroll S.A., Loh P.R. Protein-coding repeat polymorphisms strongly shape diverse human phenotypes. Science. 2021; 373:1499–1505. 34554798 10.1126/science.abg8289 PMC8549062

[85] Nelson P.T., Fardo D.W., Katsumata Y. The MUC6/AP2A2 locus and its relevance to Alzheimer's disease: a review. J. Neuropathol. Exp. Neurol. 2020; 79:568–584. 32357373 10.1093/jnen/nlaa024 PMC7241941

[86] Kumar G.R., Glaunsinger B.A. Nuclear import of cytoplasmic poly(A) binding protein restricts gene expression via hyperadenylation and nuclear retention of mRNA. Mol. Cell. Biol. 2010; 30:4996–5008. 20823266 10.1128/MCB.00600-10 PMC2953054

[87] Gharahkhani P., Burdon K.P., Fogarty R., Sharma S., Hewitt A.W., Martin S., Law M.H., Cremin K., Bailey J.N.C., Loomis S.J. et al. Common variants near ABCA1, AFAP1 and GMDS confer risk of primary open-angle glaucoma. Nat. Genet. 2014; 46:1120–1125. 25173105 10.1038/ng.3079 PMC4177327

[88] Kim D., Paggi J.M., Park C., Bennett C., Salzberg S.L. Graph-based genome alignment and genotyping with HISAT2 and HISAT-genotype. Nat. Biotechnol. 2019; 37:907–915. 31375807 10.1038/s41587-019-0201-4 PMC7605509

[89] Garrison E., Siren J., Novak A.M., Hickey G., Eizenga J.M., Dawson E.T., Jones W., Garg S., Markello C., Lin M.F. et al. Variation graph toolkit improves read mapping by representing genetic variation in the reference. Nat. Biotechnol. 2018; 36:875–879. 30125266 10.1038/nbt.4227 PMC6126949

[90] Zook J.M., Catoe D., McDaniel J., Vang L., Spies N., Sidow A., Weng Z., Liu Y., Mason C.E., Alexander N. et al. Extensive sequencing of seven human genomes to characterize benchmark reference materials. Sci. Data. 2016; 3:160025. 27271295 10.1038/sdata.2016.25 PMC4896128

[91] Ebler J., Ebert P., Clarke W.E., Rausch T., Audano P.A., Houwaart T., Mao Y., Korbel J.O., Eichler E.E., Zody M.C. et al. Pangenome-based genome inference allows efficient and accurate genotyping across a wide spectrum of variant classes. Nat. Genet. 2022; 54:518–525. 35410384 10.1038/s41588-022-01043-w PMC9005351

[92] Chiang C., Scott A.J., Davis J.R., Tsang E.K., Li X., Kim Y., Hadzic T., Damani F.N., Ganel L., GTEx Consortium et al. The impact of structural variation on human gene expression. Nat. Genet. 2017; 49:692–699. 28369037 10.1038/ng.3834 PMC5406250

[93] Yan S.M., Sherman R.M., Taylor D.J., Nair D.R., Bortvin A.N., Schatz M.C., McCoy R.C. Local adaptation and archaic introgression shape global diversity at human structural variant loci. eLife. 2021; 10:e67615. 34528508 10.7554/eLife.67615 PMC8492059

[94] Siren J., Monlong J., Chang X., Novak A.M., Eizenga J.M., Markello C., Sibbesen J.A., Hickey G., Chang P.C., Carroll A. et al. Pangenomics enables genotyping of known structural variants in 5202 diverse genomes. Science. 2021; 374:abg8871. 34914532 10.1126/science.abg8871 PMC9365333

[95] Roadmap Epigenomics C., Kundaje A., Meuleman W., Ernst J., Bilenky M., Yen A., Heravi-Moussavi A., Kheradpour P., Zhang Z., Wang J. et al. Integrative analysis of 111 reference human epigenomes. Nature. 2015; 518:317–330. 25693563 10.1038/nature14248 PMC4530010

[96] Mohamed B., Mazeaud C., Baril M., Poirier D., Sow A.A., Chatel-Chaix L., Titorenko V., Lamarre D Very-long-chain fatty acid metabolic capacity of 17-beta-hydroxysteroid dehydrogenase type 12 (HSD17B12) promotes replication of hepatitis C virus and related flaviviruses. Sci. Rep. 2020; 10:4040. 32132633 10.1038/s41598-020-61051-w PMC7055353

[97] Murga-Moreno J., Coronado-Zamora M., Bodelon A., Barbadilla A., Casillas S. PopHumanScan: the online catalog of human genome adaptation. Nucleic Acids Res. 2019; 47:D1080–D1089. 30335169 10.1093/nar/gky959 PMC6323894

[98] Zhang C., Li J., Tian L., Lu D., Yuan K., Yuan Y., Xu S. Differential natural selection of Human zinc transporter genes between African and Non-African populations. Sci. Rep. 2015; 5:9658. 25927708 10.1038/srep09658 PMC5386188

[99] Iung L.H.S., Petrini J., Ramirez-Diaz J., Salvian M., Rovadoscki G.A., Pilonetto F., Dauria B.D., Machado P.F., Coutinho L.L., Wiggans G.R. et al. Genome-wide association study for milk production traits in a Brazilian Holstein population. J. Dairy Sci. 2019; 102:5305–5314. 30904307 10.3168/jds.2018-14811

[100] Sanchez M.P., Rocha D., Charles M., Boussaha M., Hoze C., Brochard M., Delacroix-Buchet A., Grosperrin P., Boichard D Sequence-based GWAS and post-GWAS analyses reveal a key role of SLC37A1, ANKH, and regulatory regions on bovine milk mineral content. Sci. Rep. 2021; 11:7537. 33824377 10.1038/s41598-021-87078-1 PMC8024349

[101] Sabeti P.C., Varilly P., Fry B., Lohmueller J., Hostetter E., Cotsapas C., Xie X., Byrne E.H., McCarroll S.A., Gaudet R. et al. Genome-wide detection and characterization of positive selection in human populations. Nature. 2007; 449:913–918. 17943131 10.1038/nature06250 PMC2687721

[102] Ma X., Xu S. Archaic introgression contributed to the pre-agriculture adaptation of vitamin B1 metabolism in East Asia. iScience. 2022; 25:105614. 36465121 10.1016/j.isci.2022.105614 PMC9712685

[103] Zeng F., Wang Y., Kloepfer L.A., Wang S., Harris R.C. ErbB4 deletion predisposes to development of metabolic syndrome in mice. Am. J. Physiol. Endocrinol. Metab. 2018; 315:E583–E593. 29944391 10.1152/ajpendo.00166.2018 PMC6230712

[104] Mahajan A., Taliun D., Thurner M., Robertson N.R., Torres J.M., Rayner N.W., Payne A.J., Steinthorsdottir V., Scott R.A., Grarup N. et al. Fine-mapping type 2 diabetes loci to single-variant resolution using high-density imputation and islet-specific epigenome maps. Nat. Genet. 2018; 50:1505–1513. 30297969 10.1038/s41588-018-0241-6 PMC6287706

[105] Imamura M., Takahashi A., Yamauchi T., Hara K., Yasuda K., Grarup N., Zhao W., Wang X., Huerta-Chagoya A., Hu C. et al. Genome-wide association studies in the Japanese population identify seven novel loci for type 2 diabetes. Nat. Commun. 2016; 7:10531. 26818947 10.1038/ncomms10531 PMC4738362

[106] Speedy H.E., Di Bernardo M.C., Sava G.P., Dyer M.J., Holroyd A., Wang Y., Sunter N.J., Mansouri L., Juliusson G., Smedby K.E. et al. A genome-wide association study identifies multiple susceptibility loci for chronic lymphocytic leukemia. Nat. Genet. 2014; 46:56–60. 24292274 10.1038/ng.2843

[107] De Back T.R., Kater A.P., Tonino S.H. Autoimmune cytopenias in chronic lymphocytic leukemia: a concise review and treatment recommendations. Expert Rev. Hematol. 2018; 11:613–624. 29923432 10.1080/17474086.2018.1489720

[108] Gilsdorf M., Horn T., Arziman Z., Pelz O., Kiner E., Boutros M. GenomeRNAi: a database for cell-based RNAi phenotypes. 2009 update. Nucleic Acids Res. 2010; 38:D448–D452. 19910367 10.1093/nar/gkp1038 PMC2808900

[109] Aganezov S., Yan S.M., Soto D.C., Kirsche M., Zarate S., Avdeyev P., Taylor D.J., Shafin K., Shumate A., Xiao C. et al. A complete reference genome improves analysis of human genetic variation. Science. 2022; 376:eabl3533. 35357935 10.1126/science.abl3533 PMC9336181

