# Supplementary material for: Human pangenome analysis of sequences missing from the reference genome reveals their widespread evolutionary, phenotypic, and functional roles
Source: Nucleic Acids Res. 2024 Feb 14;52(5):2212–30. doi: 10.1093/nar/gkae086 (PMC10954445; doi:10.1093/nar/gkae086)
Supplement: gkae086_Supplemental_Files [file gkae086_supplemental_files.zip › Supplemental_Figures.pdf]

Human pangenome analysis of sequences missing from the reference genome reveals their widespread evolutionary, phenotypic, and functional roles

Zhikun Wu, Tong Li, Zehang Jiang, Jingjing Zheng, Yizhou Gu, Yizhi Liu, Yun Liu, Zhi Xie

Supplementary Figures

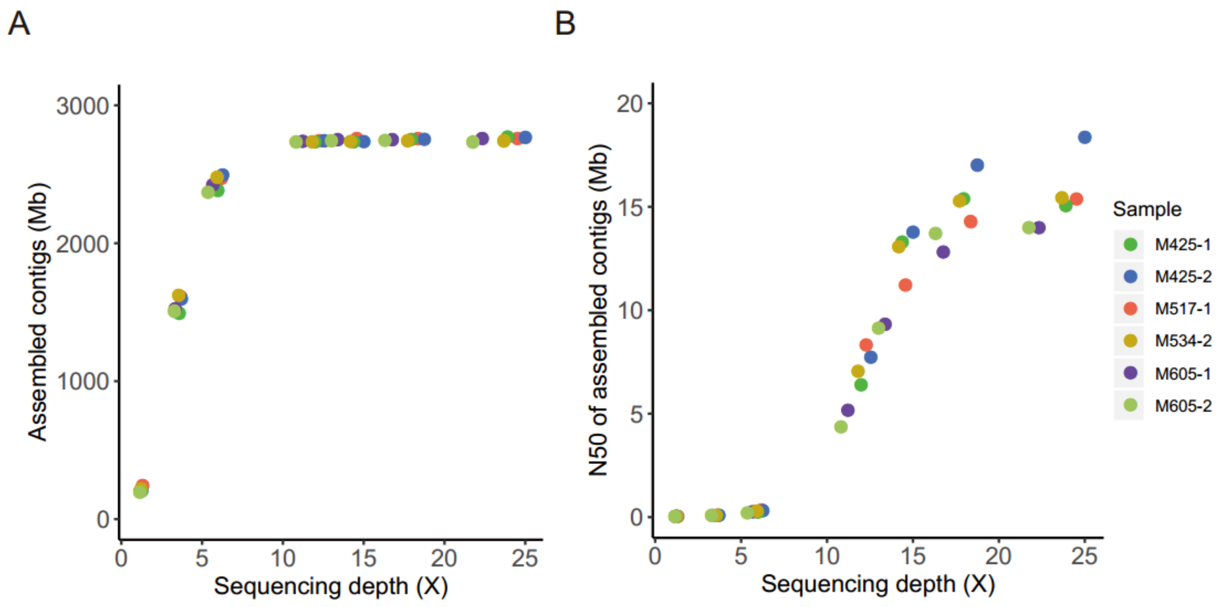

Supplementary Figure 1. Lengths of assemblies versus different sequencing depths

A, Total lengths of assemblies versus different sequencing depths.

B, N50 length of assembled contigs versus different sequencing depths.

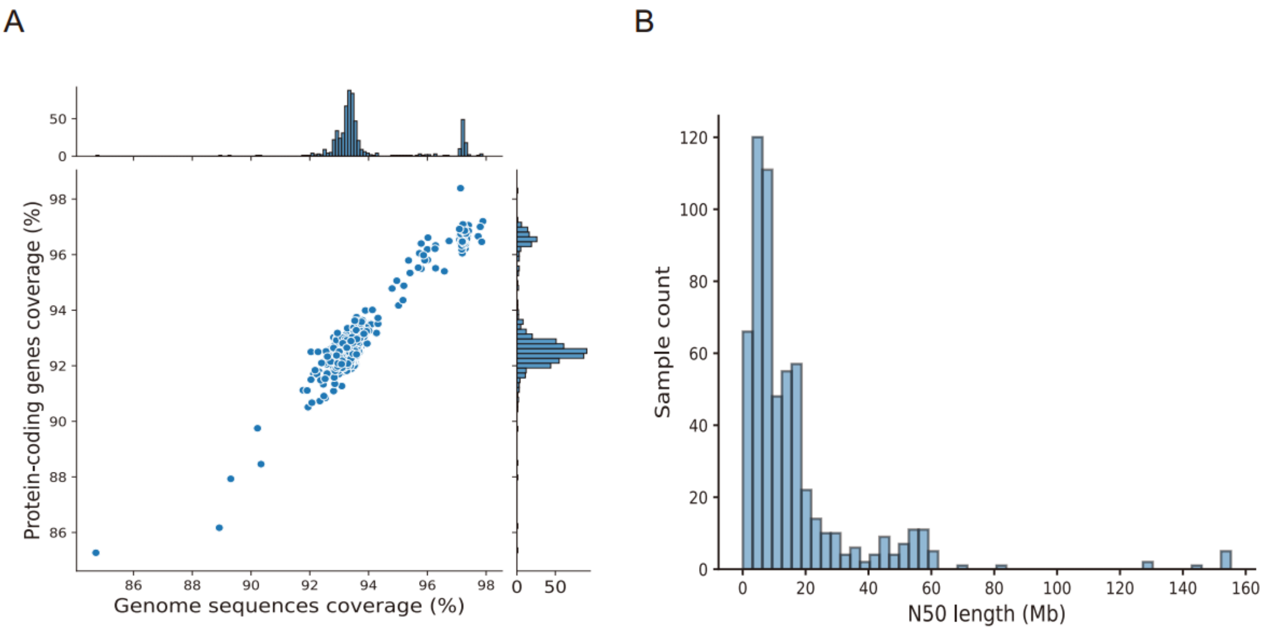

Supplementary Figure 2. Evaluation of *de novo* assembled genomes

A, Distribution of genome sequence coverage and protein-coding gene coverage.

B, Distribution of N50 length of assembled contigs.

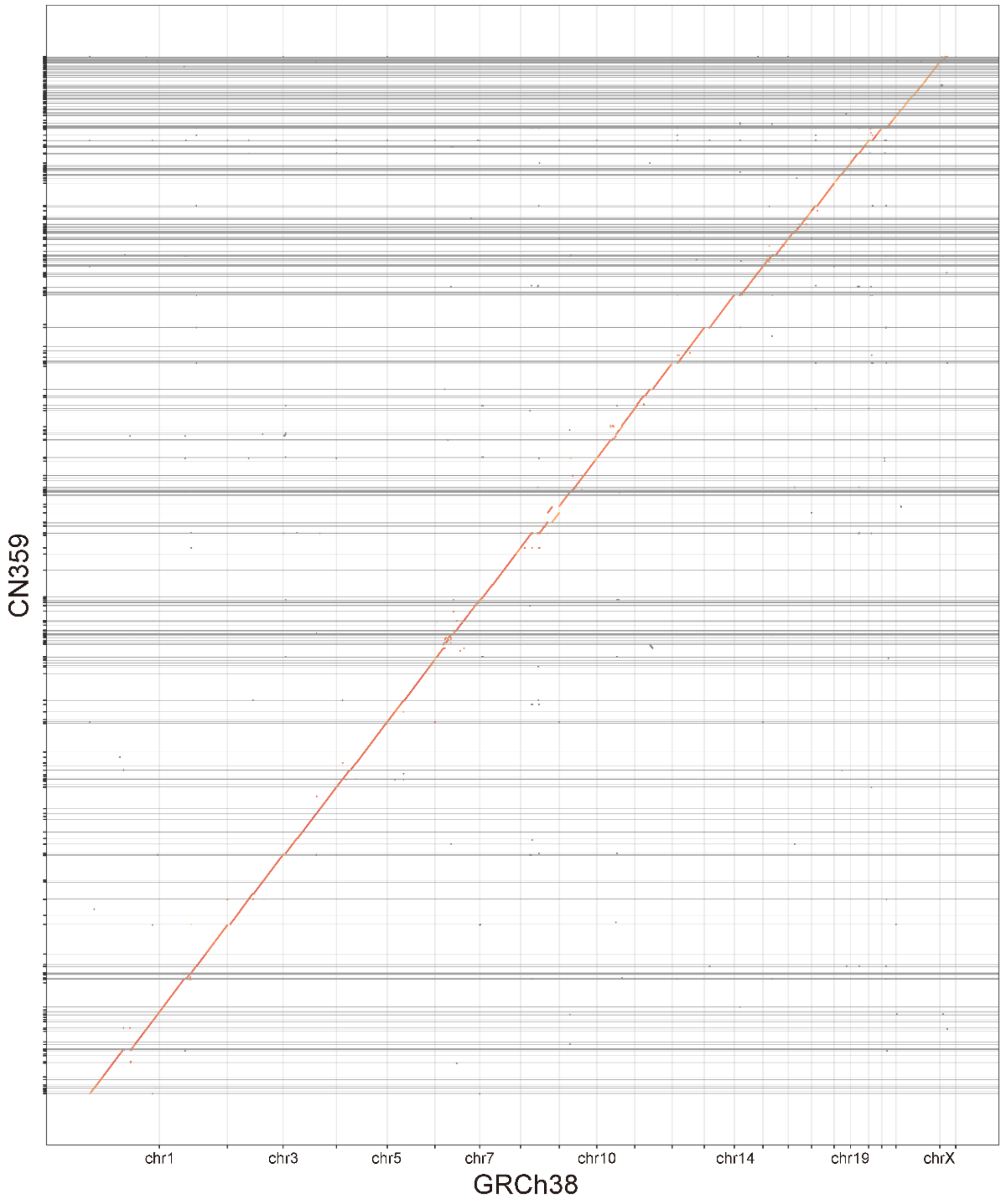

Supplementary Figure 3. Macrosynteny between assembled contigs and the reference genome CRCh38

The x and y axes are the coordinates of the reference genome GRCh38 and the assembly in this study, respectively.

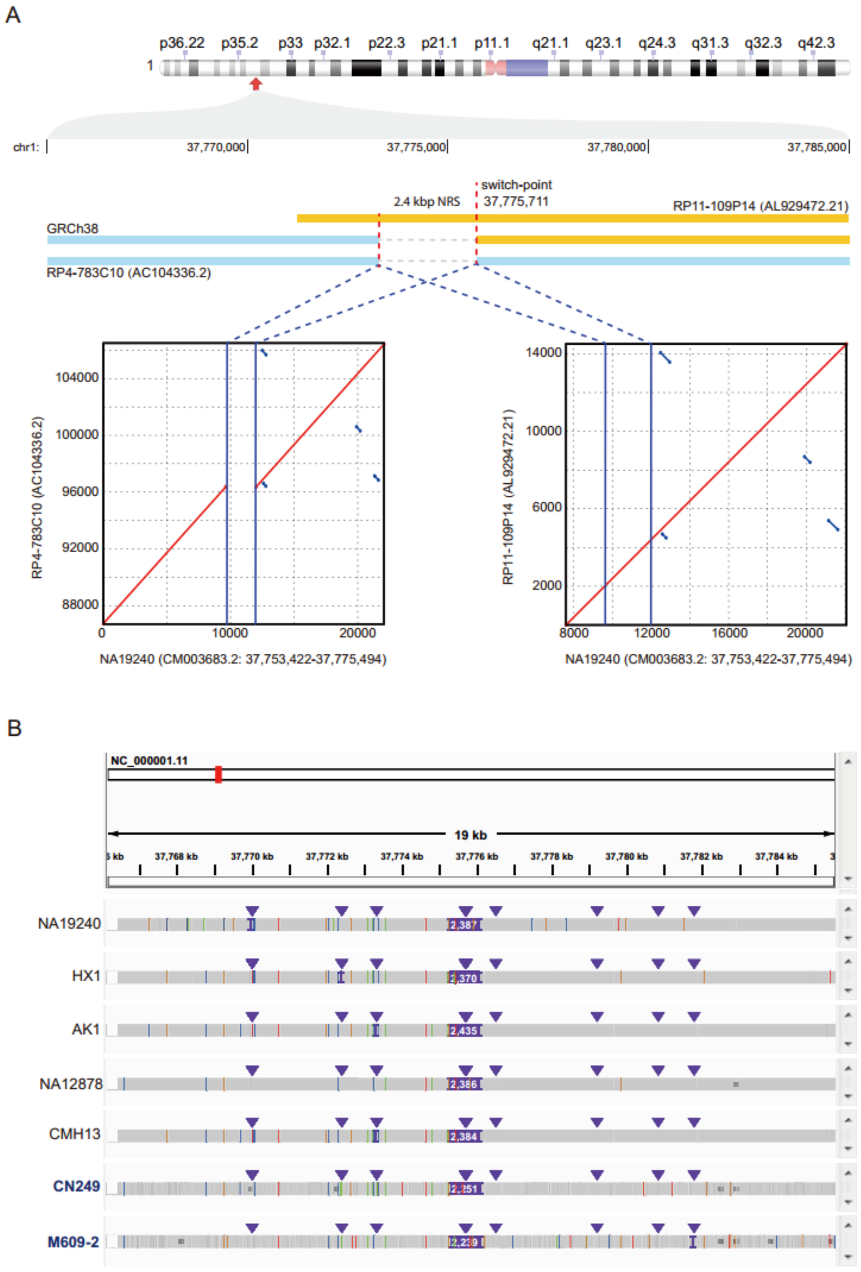

Supplementary Figure 4. An example of mis-assembly from GRCh38

A, A 2.4 kb NRS was precisely anchored at the switch-point of BACs RP4-783C10 (AC104336.2) and RP11-109P14 (AL929472.21). Compared to NA12940 (x-axis), the deleted sequence of RP4-783C10 (AC104336.2) (y-axis) resulted in a missing sequence in final assembly of GRCh38 in the switch-point of these two BACs.

B, The diagram shows that several published genomes contain a 2.4 kb NRS at the switch-point of GRCh38. The sample name in blue indicates genome assembly generated in this study.

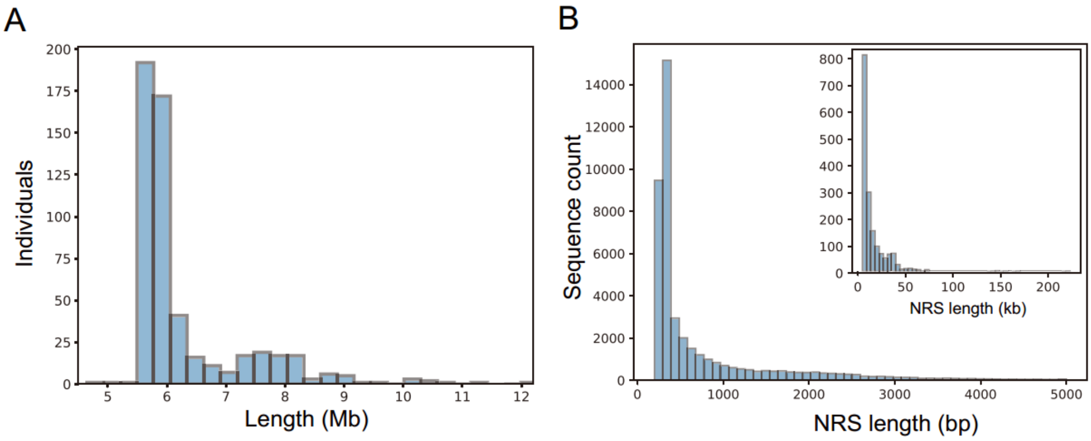

Supplementary Figure 5. Length distribution of the NRSs

A, Length distribution of the extracted NRSs for the 539 *de novo* assembled genomes.
B, Length distribution of the non-redundant NRSs for the whole population.

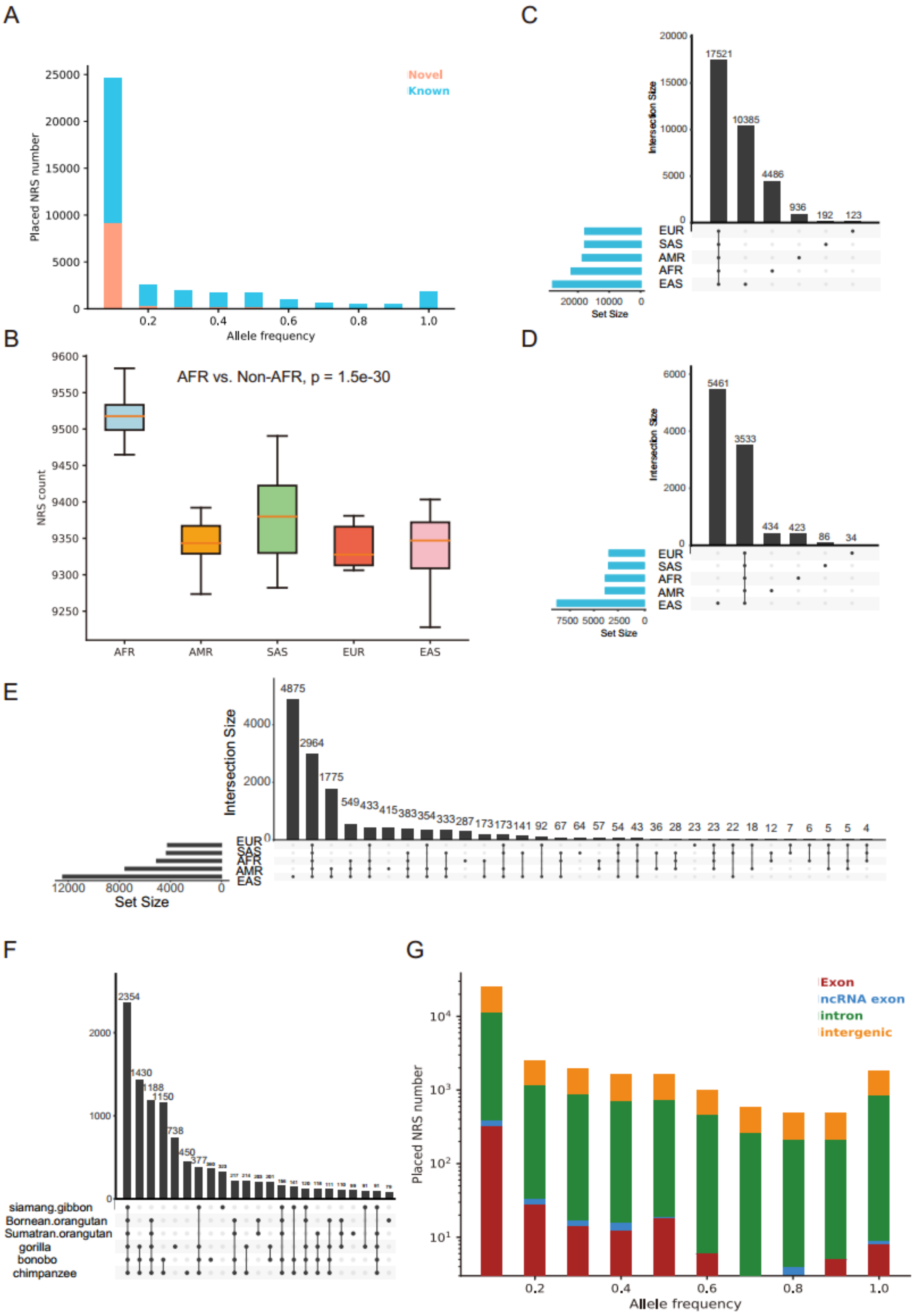

Supplementary Figure 6. Characterization of NRSs for the whole population

A, Allele frequency of the non-redundant NRSs.

B, Boxplots show the NRS counts for different populations. The NRS counts across different platforms were normalized using z-score. AFR: African, AMR: American, SAS, South Asian, EUR, European, EAS, East Asian. The center line in the box

indicates the median, the lower and upper hinges indicate the first and third interquartile range (IQR). The lower and upper whiskers show the values greater than 25th quartile minus 1.5 × IQR and less than 75th quartile plus 1.5 × IQR, respectively. Where data beyond these ranges are shown as individual points.
C, Overview of shared and distinctive NRSs across diverse populations before excluding NRSs from Chinese Pangenome Consortium (CPC).
D, Overview of shared and distinctive NRSs across diverse populations after excluding NRSs from CPC.
E, The number of novel NRSs across diverse populations.
F. The number of overlapped NRSs among six non-human primate genomes.
G, The gene feature annotation of placed NRSs with different allele frequencies.

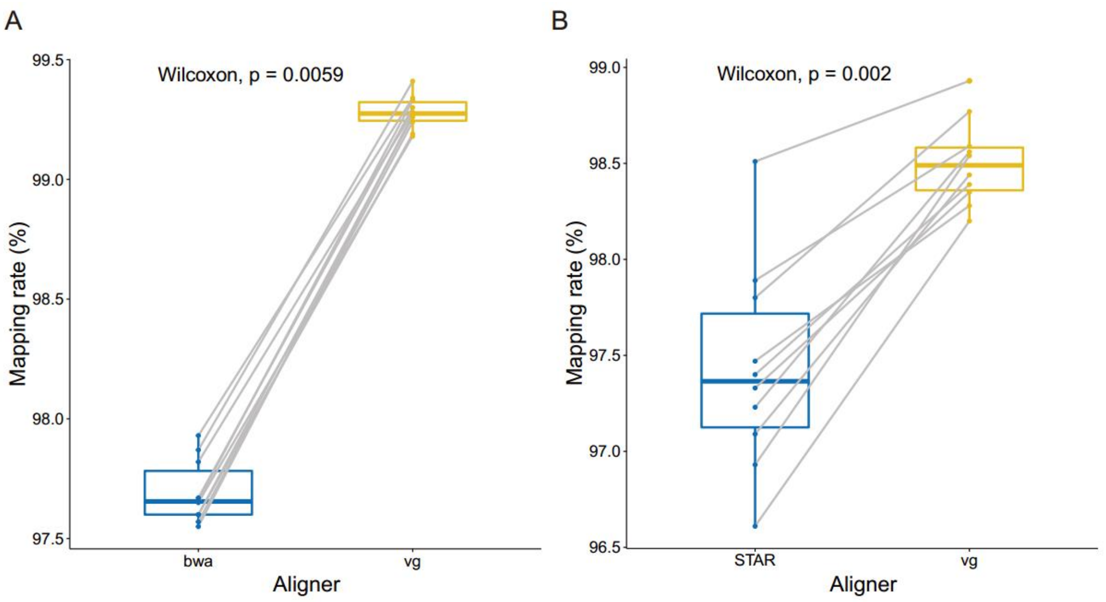

Supplementary Figure 7. Mapping rate improvement of short-read sequencing data

A, The mapping rate improvement of DNA from short-read sequencing platform, bwa and vg are aligners against the conventional linear genome GRCh38 and graph pangenome in this study, respectively.

B, The mapping rate improvement of RNA from short-read sequencing platform, STAR and vg are aligners against the conventional linear genome GRCh38 and graph pangenome, respectively.

Wilcoxon signed-rank test was conducted for the mapping rates of ten samples.

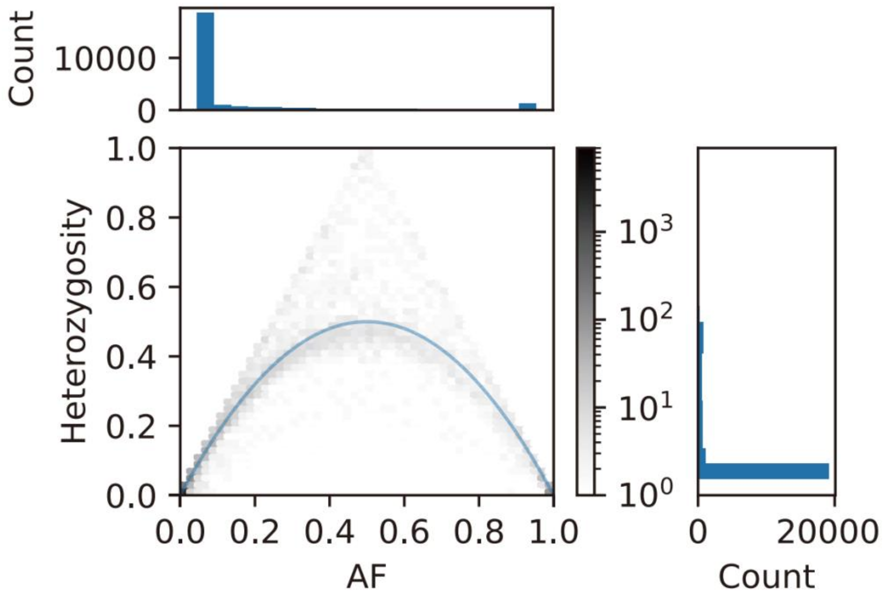

Supplementary Figure 8. Genotyping information of NRSs

The plot shows the relationship between allele frequency (AF) and heterozygosity of the genotypes of NRSs derived from the graph pangenome.

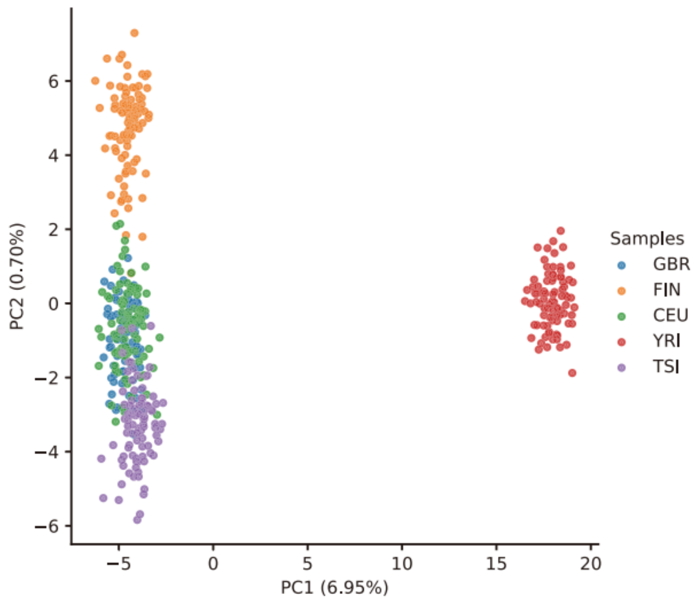

Supplementary Figure 9. Population stratification analysis based on genotypes of NRSs

The principal component analysis (PCA) based on NRSs from short-read sequencing data detects population stratification for Genetic European Variation in Disease (GEUVADIS) consortium, which consist of four European-ancestry and one African-ancestry populations. GBR: British, FIN: Finnish, CEU: Utah residents (CEPH), TSI: Toscani, YRI: Yoruba. The values in parentheses indicate the genetic variations explained by the first two PCs.

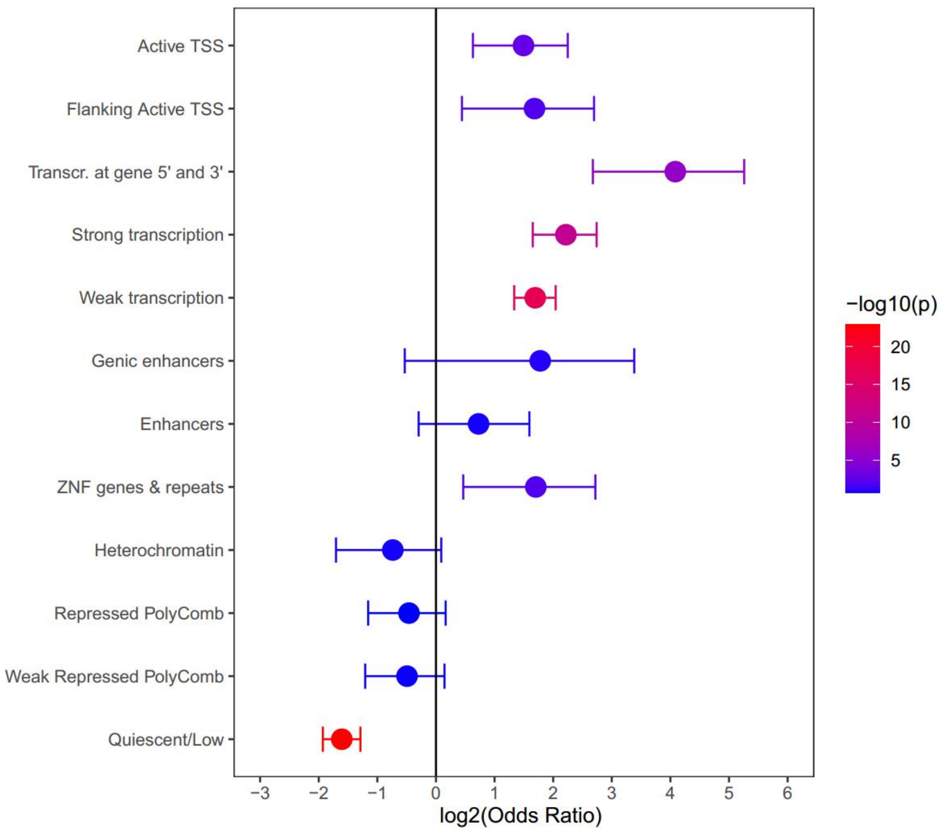

Supplementary Figure 10. Enrichment or depletion of eQTL-associated NRSs

The enrichment or depletion of eQTL-associated NRSs that intersected with the epigenetic states by the Roadmap Epigenetics Consortium (REC). The Fisher's exact test was conducted, and p values were corrected using Benjamini-Hochberg method.

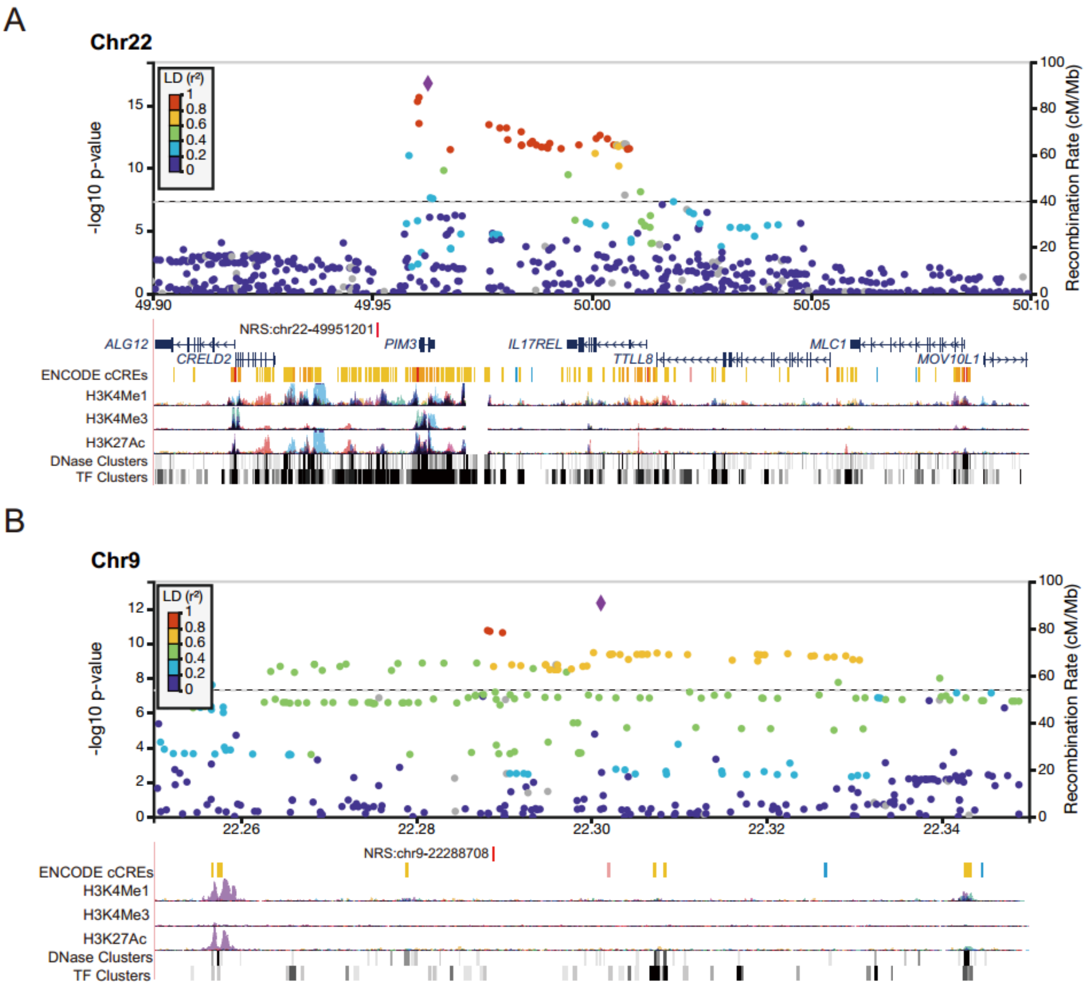

Supplementary Figure 11. The regional manhattan plot for SNPs associated with diabetes.

A, The top signal was significantly associated with diabetes ($P = 1.8\times10^{-17}$, Mahajan et al., 2018). The NRS is 9.6 kb upstream of *PIM3* and intersected with H3K27Ac, H3K4Me1 and TF clusters.

B, The top signal was significantly associated with diabetes ($P = 4.7\times10^{-13}$, Imamura et al., 2016). There are three SNPs in high LD ($r^2 > 0.8$) with top signal of diabetes. And the NRS (red vertical line) located in the region of three SNPs.

The pairwise r^2 values were from the 1000 Genome Phase3 (EAS) reference panel.

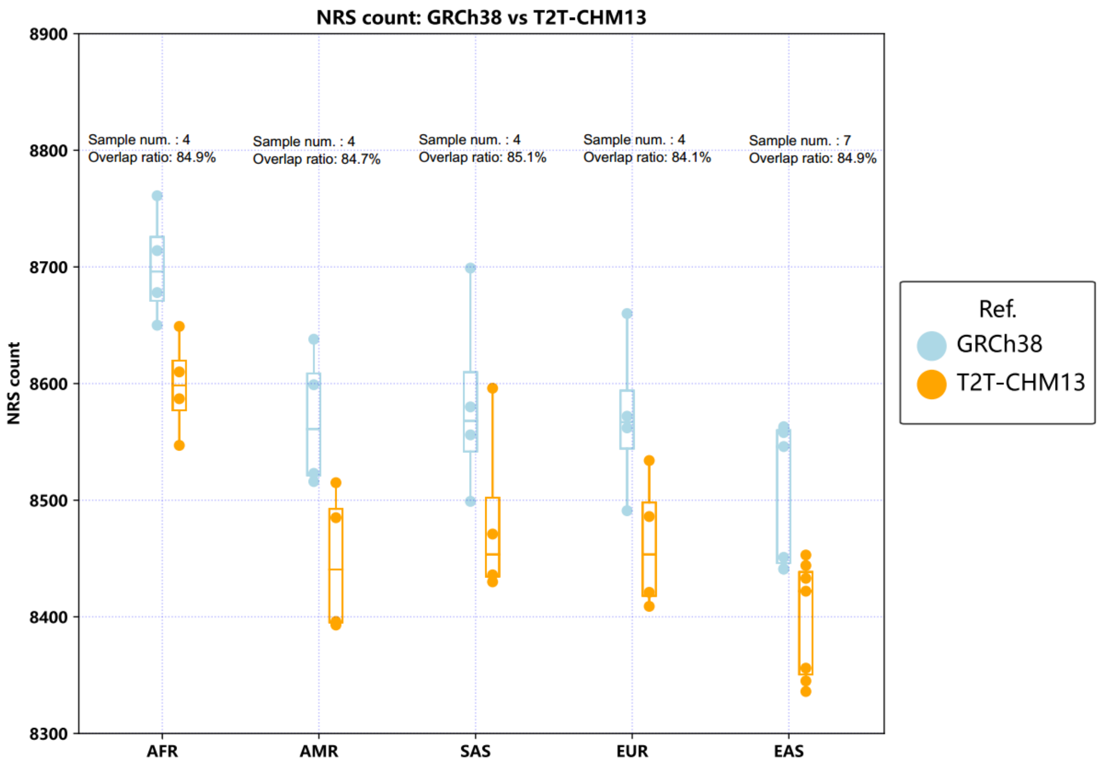

Supplementary Figure 12. The number of placed NRSs identified based on the references of T2T-CHM13 and GRCh38

Boxplots show the NRS counts for different populations. The NRS counts across different platforms were normalized using z-score. AFR: African, AMR: American, SAS: South Asian, EUR: European, EAS: East Asian. The center line in the box indicates the median, the lower and upper hinges indicate the first and third interquartile range (IQR). The lower and upper whiskers show the values greater than 25th quartile minus 1.5 × IQR and less than 75th quartile plus 1.5 × IQR, respectively. Where data beyond these ranges are shown as individual points.
